# Biocontrol, plant growth-promoting, and bioremediation potential of *Aeromonas veronii* CMF from the gut of *Chrysomya megacephala*

**DOI:** 10.1128/spectrum.01622-25

**Published:** 2025-11-04

**Authors:** Sandipan Banerjee, Kunal Kumar Saha, Krishnendu Pramanik, Raju Biswas, Moumita Parveen, Srinivasan Balachandran, Hynek Roubík, Narayan Chandra Mandal

**Affiliations:** 1 Mycology and Plant Pathology Laboratory, Department of Botany, Visva-Bharati 449338 , Santiniketan, West Bengal, India; 2 Faculty of Forestry and Wood Sciences, Czech University of Life Sciences Prague 206518 , Prague, Czech Republic; 3 Department of Sustainable Technologies, Faculty of Tropical AgriSciences, Czech University of Life Sciences Prague 654350 , Prague, Czech Republic; 4 Department of Botany, Cooch Behar Panchanan Barma University 532541, Cooch Behar, West Bengal, India; 5 Microbiology Laboratory, Department of Botany, Visva Bharati 449338 , Santiniketan, West Bengal, India; 6 Ecosystems Laboratory, Centre for Ecological Sciences (CES), Indian Institute of Science 29120, Bengaluru, Karnataka, India; 7 Bioenergy Laboratory, Department of Environmental Studies, Institute of Science, Visva-Bharati 449338 , Santiniketan, West Bengal, India; Instituto de Ecología, A.C. (INECOL), Pátzcuaro, Michoacán, Mexico

**Keywords:** antifungal activity, chitinase, β-glucanase, protease, insect gut symbiont, heavy metal, plant-microbe interaction, root colonization

## Abstract

Gut system of chitinivorous insect *Chrysomya megacephala* was purposefully targeted to find unexplored microbial resources based on the rationale of their usual food habits. A phytobeneficial bacterial strain *Aeromonas veronii* CMF was successfully isolated and characterized up to genomic approaches. The gut isolate *A. veronii* CMF is a non-pathogenic strain, as proven by the negative results from the hemolysis and DNase tests. Antifungal enzyme production by the CMF exhibited 22.14 ± 2.12, 16.09 ± 0.476, and 1.89 ± 0.46 U/mL chitinase, protease, and β−1,3-glucanase production, respectively. Further, *in vitro* and *in vivo* studies also elucidate the effective utilization of such gut bacterial attributes against as many as nine plant pathogenic fungi, demonstrating plant growth-promoting (PGP) and root-colonizing activities with *Cicer arietinum* and *Oryza sativa* IR36, as well as heavy metal(loid)s (HMs) resistance, removal, and bioaccumulation potential. Hence, the current study revealed the potential of the gut symbiont CMF to respond against both the biotic and abiotic stresses with PGP attributes for sustainable agriculture.

**IMPORTANCE:**

Gut symbiont *A. veronii* CMF, with integrated antifungal (chitinase, protease, and β−1,3-glucanase activity), plant growth-promoting (including plant root colonizing potential), and bioremediational attributes can be harnessed as a biotechnological tool for sustainable agriculture and human welfare by fulfilling several sustainable developmental goals. On the basis of such multidimensional gut symbiotic attributes which are validated through genomic-phenotypic observations during this study, it can be suggested that this gut symbiont can perform the host beneficial attributes in the plant rhizosphere, i.e., the “plant gut system” and consequently act as “plant gut symbionts.”

## INTRODUCTION

Global food crisis is one of the discernible situations that necessitates its substantial attention due to high population growth with a proportionate decrease in cultivable land, reduction in crop productivity through pathogenic interference, and environmental pollution (1). The destruction of agronomic crops due to the incursion of phytopathogenic fungi is a challenging global issue (2). Biocontrol agents, found in close association with plants, antagonize phytopathogens mainly by producing fungal cell-wall lytic enzymes that naturally suppress further pathogen proliferation within plants. Antifungal enzymes (chitinases, proteases, and β-glucanases) are widely recognized as cell-wall lytic enzymes, since fungal cell walls are primarily composed of chitin, proteins, and β-glucan. However, varied bioactive metabolites are also responsible for antifungal activity, including organic acids, antimicrobial peptides, bacteriocins, polyketides, polyenes, macrolides, aminoglycosides, nucleosides, cyclic organic compounds, and phenolic compounds (3, 4).

Extracellular enzymatic activity of the gut microbial association for dietary digestion is considered one of the indispensable criteria for the selection and establishment of gut microbial symbiosis in any gut system (5). In addition, the gut system functions as a continuous culture platform where autochthonous microbes capable of degrading dietary constituents are retained and proliferate, whereas allochthonous microbes lacking this ability are eliminated (6, 7). Although the host gut systems can digest the food materials by breaking them into smaller units, symbionts simultaneously accelerate the completion of digestion by transforming these smaller molecules into the assimilatory state through their enzymatic activities (8, 9). Beyond their enzymatic role in digestion, these symbionts are also recognized for conferring multiple host beneficial activities, such as pathogen and pest biocontrol (10), production of antimicrobial compounds (11), detoxification of plant defense compounds and insecticides (12), and bioremediation (13), as well as providing vitamins, amino acids, and lactic acids to their hosts (14, 15). In this context, the gut system associated with chitinaceous and proteinaceous diet can be a viable option for the exploration of chitinolytic and proteolytic microorganisms, which may be harnessed as a potent biocontrol agent due to their enzymatic characteristics. Moreover, microbial representatives of catalytic biomolecules with bacterial origin are in high demand in biotechnology because of their high catalytic efficiency, short processing time, low energy consumption, cost-effectiveness, biochemical flexibility, and potential for manipulation through recombinant DNA techniques, protein engineering, and eco-sustainable practice (16, 17).

Gut systems with chitinaceous and proteinaceous diets are commonly observed among insects (18). Insects are the most diversified species-rich groups of invertebrates having ecological niches (19). In these circumstances, the nutritional fortification offered by the gut symbionts to insects is considered strategic to the evolutionary success of this assemblage (20). To achieve such ecological adaptability, gut symbionts play a fundamental role in assisting host digestion via enhanced extracellular digestive enzyme production, progressive digestive aptitudes on suboptimal diets, and rapid shifts in community structure in response to dietary changes (21, 22). Despite the existence of around one million insect species, grouped into 31 orders, only a few reports are available regarding the biocontrol potential of their gut symbionts. In that scenario, *Chrysomya megacephala* (Diptera) appears as one of the unexplored gut systems. In case of nutritional enrichment, *C. megacephala* primarily feeds on the animal carcass and fish scales, which contain chitin, β-glucan, and protein (23). Depending upon their feeding habits, these gut systems can be considered as promising sources of potent chitinase- and protease-producing gut symbionts. To meet the global demand for effective biocontrol agents, such gut systems with potential bacterial representatives may serve as novel resources, as these microbes have undergone diverse selection pressures within host gut environments for the establishment of gut-symbiotic relationships. Several PGP traits, such as IAA production (24, 25), phosphate solubilization (26), siderophore production (27, 28), biofilm formation (29), and nitrogen (N_2_) fixation (30), as well as bioremediating attributes (31, 32) have also been documented, though largely in a host-specific context.

Although recent studies have highlighted gut bacterial capabilities, ranging from plant pathogens biocontrol to bioremediation (33), extensive genetic classification of these enzymes remains lacking, particularly for antifungal enzymes like chitinase. Given their considerable biotechnological potential, physicochemical characterization, secondary structural analysis, and homology-based protein modeling of gut symbionts derived from different chitinase and proteases could uncover new opportunities for protein engineering (e.g., enhancing catalytic efficacy, improved thermal stability) and genetic manipulation (e.g., antifungal enzyme producing recombinant strain development with exceptional specificity). Such advances may facilitate large-scale industrial production of antifungal enzyme production (34).

In recent decades, the agricultural economy has faced both quantitative and qualitative losses due to biotic (pests and pathogens) and abiotic (climatic changes and hazardous materials contamination) stresses during both pre- and post-harvest phases (35, 36). Among abiotic stresses, heavy metal (HM) contamination in agricultural soil has emerged as a major environmental challenge, reducing crop quality and productivity, and threatening human health through food chain bioaccumulation (37, 38). Similarly, intensive crop management practices have involved excessive application of chemical pesticides, fungicides, and fertilizers by compromising environmental stability, affecting public health, and inducing pathogen resistance to synthetic agents. Consequently, researchers worldwide are seeking unexplored microbial resources as an eco-sustainable and green strategy to ensure crop productivity (39). In this regard, PGP gut bacteria with bioremediating potential against agricultural pollutants [heavy metal(loid)s, polycyclic aromatic hydrocarbons, pesticides, and fungicides] are gaining attention (31, 40) for their role in minimizing bioaccumulation of such hazards. Thus, gut microbiota may serve as a golden reservoir to mitigate both biotic and abiotic stresses in agricultural ecosystems.

Therefore, keeping all these gut symbiotic relationships in mind, exploration of such chitinaceous and proteinaceous food-consuming gut systems of *C. megacephala* can be harnessed as a potential biotechnological tool for human welfare. In this regard, potential gut symbionts may serve as novel integrated plant growth-promoting agents for managing biotic and abiotic stresses in sustainable agronomic crop biology, offering an alternative to conventional hazardous chemical fungicides, insecticides, and fertilizers.

## RESULTS AND DISCUSSION

### Isolation and identification of insect and gut bacteria CMF

The selected insect was identified as *Chrysomya megacephala* (Order-Diptera), from the Zoological Survey of India, Kolkata. Isolated gut symbiont was identified as *Aeromonas veronii* CMF. Isolation and identification of the gut bacteria were described in Bhattacherjee et al. (18). The NCBI details are as follows: (1) GenBank Accession No. WVRP00000000; (2) BioProject ID: PRJNA596393; and (3) BioSample ID: SAMN13631568. In addition, reports are available on *A. veronii* as a plant growth-promoting bacteria from versatile resources, ranging from the rhizosphere (41) to aquaponics (42).

#### Pathogenicity test of the gut isolate CMF

The gut isolate *A. veronii* CMF exhibited a non-pathogenic nature, which was confirmed by negative results from hemolysis assay (Fig. S1A) and DNase test (Fig. S1B). However, pathogenic reference strain *Bacillus subtilis* MTCC 121 showed its pathogenic potential in both assays, as evidenced by the formation of halo zone in hemolysis assay (Fig. S1A) as well as in the DNase test (Fig. S1B). A similar non-pathogenic nature of *A. salmonicida* was also observed by Tewari et al. (43), whereas the pathogenic nature of *B. subtilis* MTCC 121 was also reported by Biswas et al. (44).

### Antifungal enzyme-producing potential of CMF

**Fig 1:**
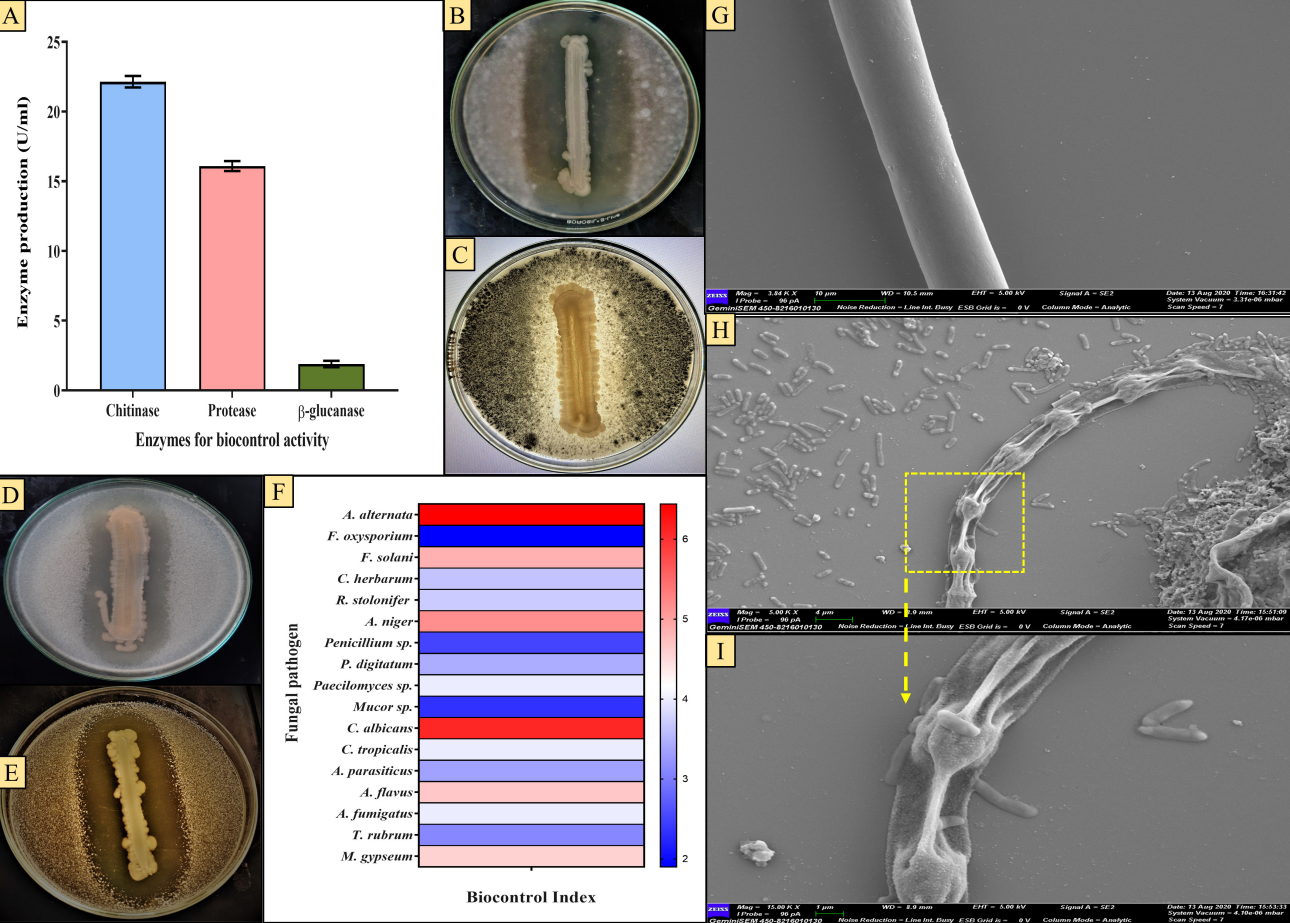
Biocontrol potential of the gut isolate *A. veronii* CMF. (**A**) antifungal enzyme production by CMF, (**B–E **) Zone of inhibition against plant and animal pathogens: (**B**) *A. alternata*, (**C**) *A. niger*, (**D**) *F. solani*, (**E**) *C. albicans,* and (**F**) biocontrol Index of CMF. (**G–I**) SEM analysis of biocontrol potential of CMF: (**G**) healthy mycelium of *A. alternata* and (**H and I**) deformed mycelia of *A. alternata* co-cultured with *A. veronii* CMF.

The gut isolate *A. veronii* CMF showed potential enzyme production for all fungal cell-wall lytic enzymes tested, i.e., chitinase, protease, and β−1,3-glucanase. Qualitative assay confirms the production of all three antifungal enzymes by CMF, indicated by clear halo zone formation around the bacterial colony using the respective substrates (Fig. S2A through C). In the case of chitinase and β−1,3-glucanase production ability by CMF, the gut bacteria produce 22.14 ± 2.12 and 1.89 ± 0.46 U/mL of chitinase and β−1,3-glucanase, respectively (Fig. 1A) (Table S1), while protease production potential of CMF was documented as 16.09 ± 0.476 U/mL, as described in our previous report (Bhattacherjee et al. [18]). In this connection, there is no such report from *A. veronii* or from other *Aeromonas* species for such antifungal enzymatic potential especially from gut isolates.

#### Determination of the nature of antifungal metabolites

Antifungal activity was observed only from the CFS of *A. veronii* CMF, showing a prominent zone of inhibition surrounding the well (W1) against pathogenic fungi *A. alternata* VBAV007. No zone of inhibition or antifungal activities was observed surrounding the wells which contained heat-killed CFS (W2), proteinase K-treated CFS (W3), and control CFS (W4) (Fig. S3A and B). Such observations suggested that the antifungal metabolites produced in the CFS of *A. veronii* CMF are not thermostable and proteinaceous in nature. Moreover, positive outcomes from qualitative (clear halo zone formation) and quantified enzyme assay also strongly confirmed prominent antifungal enzymatic (chitinase, protease, and β-glucanase) potential of *A. veronii* CMF (Fig. S2A through C). Comparable results were also documented by Gorai et al. (45).

### *In silico* characterization of chitinases from CMF

#### Physicochemical characterization of the chitinases from *A*. *veronii* CMF genome

Genetic insight of the *A. veronii* CMF genome revealed a total of seven chitinases: three different GH 18 family chitinases (locus tag = GS629_17520, GS629_10520, and GS629_07790), two different GH 19 family chitinases (locus tag = GS629_07780 and GS629_20720), one chitobiase (locus tag = GS629_12090), and one chitin disaccharide deacetylase (locus tag = GS629_08665). Table 1 lists all the analyzed physicochemical features (amino acid length, molecular weight, isoelectric point, extinction coefficient, instability index, aliphatic index, GRAVY value, Tm index, and Tm value).

**TABLE 1:** Characterization of chitinases from *A. veronii* CMF genome

Protein/NCBI GenBank Acc. no./PMDB ID	Physicochemical characteristics									Secondary structure prediction		
	Amino acid length	Molecular weight (KDa)	Isoelectric point (pI)	Extinction coefficient (EC) (M^−1^ cm^−1^)	Instability index (II)	Aliphatic index (AI)	GRAVY value	Tm index	Tm value	Helix (%)	Sheet (%)	Turn (%)
GH 18 family chitinase/MXV30556.1/PM0084140	998	106.54	5.51	226,145	28.08	71.24	−0.299	0.846	55°C~65°C	56.7	31.2	14.9
GH 18 family chitinase/MXV29181.1/PM0084138	728	78.83	4.63	140,525	29.47	74.60	−0.356	0.476	55°C~65°C	76.8	34.3	13.4
GH 18 family chitinase/MXV28648.1/PM0084136	866	92.74	5.11	188,535	23.52	75.29	−0.353	0.644	55°C~65°C	61.0	52.2	12.4
GH 19 family chitinase/MXV28646.1/PM0084135	638	69.78	5.10	152,805	27.25	62.29	−0.522	0.699	55°C~65°C	82.0	64.2	11.8
GH 19 family chitinase/MXV31165.1/PM0084141	483	53.26	6.21	142,375	27.26	66.56	−0.404	0.576	55°C~65°C	68.5	58.8	13.7
Chitobiase/MXV29488.1/PM0084139	577	64.57	5.57	125,875	40.12	74.38	−0.445	−0.38	<55°C	60.2	39.3	14.1
Chitin disaccharide deacetylase/MXV28820.1/PM0084137	254	28.55	5.64	32,680	37.63	94.17	−0.197	0.257	55°C~65°C	60.9	57.2	14.3

#### Secondary structure prediction of the chitinases from *A*. *veronii* CMF genome

Secondary structure prediction analysis indicates that chitinases from the *A. veronii* CMF genome exhibit α-helix arrangements ranging from 56.7% to 82.0% (Table 1). Additionally, the *A. veronii* CMF genome displays β-sheet arrangements with a range of 31.2% to 64.2% (Table 1). The analysis also identifies β-turn arrangements in the chitinases, ranging from 11.8% to 14.9% (Table 1). This analysis reflects the stable nature of all the studied proteins. To the authors’ knowledge, similar *in silico* analyses of bacterial chitinases, particularly those from the GH 18 and 19 families, chitobiase, and chitin disaccharide deacetylase, have not been reported.

#### Homology protein modeling, structural assessment, validation, and submission

##### Homology protein modeling of the chitinases from *A*. *veronii* CMF genome

After the target vs template alignment, homology protein model was built using the best-matching template (Table S3) obtained from SWISS workspace. The built model (A = cartoon view; B = surface view) reflects the findings of predicted secondary structural conformations, helix, sheet, and loop, which are indicated in red, yellow, and green, respectively. The homology protein model or 3D model of all seven antifungal chitinases, i.e., MXV30556.1 (Fig. S4A and B), MXV29181.1 (Fig. S5A and B), MXV28648.1 (Fig. S6A and B), MXV28646.1 (Fig. S7A and B), MXV31165.1 (Fig. S8A and B), MXV29488.1 (Fig. S9A and B), and MXV28820.1 (Fig. S10A and B), was submitted to the protein model database (PMDB) for further studies (Table S4).

##### Structural assessment and validation of the chitinases from *A*. *veronii* CMF genome

In this evaluation, as a part of the structural assessment, Z-score and QMEAN analyses depict that among the seven chitinases, GH 18 family chitinases MXV30556.1, MXV29181.1, and MXV28648.1 exhibited Z-scores and QMEAN scores of <1 and 0.26 (Fig. S4C), <1 and 0.74 (Fig. S5C), and <1 and −0.53 (Fig. S6C), respectively. On the other hand, GH 19 family chitinases MXV28646.1 and MXV31165.1 revealed Z-scores and QMEAN scores <1 and −3.13 (Fig. S7C) and <1 and −5.65 (Fig. S8C), respectively, whereas, chitobiase (MXV29488.1) and chitin disaccharide deacetylase (MXV28820.1) display Z-scores and QMEAN scores of <1 and −3.03 (Fig. S9C) and <1 and −1.88 (Fig. S10C), respectively.

In the next step, the Ramachandran plot was used to validate the distribution of amino acid residues in the most favored regions. The results showed that >89.7% (Fig. S4D and E), >88.5% (Fig. S5D and E), >90.2% (Fig. S6D and E), >87.3% (Fig. S7D and E), >76.1% (Fig. S8D and E), >90.6% (Fig. S9D and E), and >87.5% (Fig. S10D and E) of residues were in the most favored regions for GH 18 family chitinases (MXV30556.1, MXV29181.1, and MXV28648.1), GH 19 family chitinases (MXV28646.1 and MXV31165.1), chitobiase (MXV29488.1), and chitin disaccharide deacetylase (MXV28820.1), respectively. In most cases, these data significantly satisfy the criteria for a good quality model (>90%) (46).

Further, overall quality factor determined using the SAVES ERRAT tool was 94.165 (Fig. S4F), 91.734 (Fig. S5F), 91.296 (Fig. S6F), 95.161 (Fig. S7F), 74.590 (Fig. S8F), 88.628 (Fig. S9F), and 84.669 (Fig. S10F) for GH 18 family chitinases (MXV30556.1, MXV29181.1, and MXV28648.1 ), GH 19 family chitinases (MXV28646.1 and MXV31165.1), chitobiase (MXV29488.1), and chitin disaccharide deacetylase (MXV28820.1), respectively. Most of the time, these data can considerably fulfill the requirements of a score of 90% or higher as an overall quality factor for a model of good, high-resolution structure (47).

### Phylogenetic study

Evolutionary study using the built phylogenetic tree revealed the formation of two distinct groups (Fig. S11). It is evident from the tree that GH18 and GH19 family chitinases formed a united cluster, whereas an outgroup of chitobiase and chitin disaccharide deacetylase was also established (Fig. S11). This analysis suggests a close evolutionary relatedness among GH18 and GH19 family chitinases; however, there may be a bit of evolutionary distance among the other proteins indicated in the outgroups (MXV29488.1 and MXV28820.1). However, from a functional point of view, these enzymes found in the *Aeromonas veronii* CMF genome are commonly associated as potential antifungal enzymes. Further investigation is required to decipher the distinctive characteristics of the studied proteins of interest that may direct some novel structural information concealed therein for biotechnological applications.

### Functional analysis

STRING analyses have revealed functional interacting partners of six out of seven antifungal enzymes (Fig. S12 to S17) of CMF genome. Based on sequence identity, bitscore, and e-value, the best-matched protein was selected to establish the protein-protein interaction network for each protein. It was found that MXV28648.1 (glycoside hydrolase) of the CMF genome was 92.1% identical to the AMQ41956.1 protein in the STRING database, while MXV29181.1 (DUF5011 domain-containing protein) shared 98.1% identity with AMQ41023.1 from *Aeromonas veronii* (Fig. S12 and S13). Similarly, MXV30556.1 (chitinase) and MXV28646.1 (chitinase) showed 62.3% and 98.1% identities with AMQ41023.1 and AMQ41957.1, respectively (Fig. S14 and S15). In the case of the other two proteins, viz., MXV31165.1 (carbohydrate-binding protein) showed only 47.6% identity with AMQ41957.1 (Fig. S16); however, MXV29488.1 (chitobiase) matched only with AMQ42136.1, providing no exact identity information for this protein in the STRING database (Fig. S17). The list of predicted functional partners is available in Fig. S12 to S17 against each protein. This network is an important tool for deciphering system-level cellular processes (34), whereas it provides intuitional information on the evolutionary aspects of a protein of interest apart from its structure-function relationship (48).

### Biocontrol potential of CMF against fungal pathogens

Mycolytic enzyme production, particularly the cell-wall lytic enzymes such as chitinases, proteases, and β-glucanases, is a phenomenal characteristic of any potential biocontrolling agent, along with other therapeutic biomolecules (49). Following its antifungal enzyme-producing capabilities, the gut isolate *A. veronii* CMF also exhibited notable antagonistic activity (Fig. 1B through E) as well as biocontrol index (Fig. 1F) against all the tested pathogenic fungi (Table S5). Here, *A. veronii* CMF exhibited utmost activity against *A. alternata* VBAV007. In SEM micrograph examinations, prominent degradation and deformation of the selected pathogenic fungal mycelial structures were observed. The antagonistic effect of *A. veronii* CMF on *A. alternata* VBAV007 (Fig. 1G through I) was reflected in such a way that massive deconstruction along the gut bacterial cells, specifically on the cell wall morphologies, was observed in comparison to the healthy mycelia of the control set. These morphological observations intensely suggest that the mycelial disintegration results from the cell wall-lytic antifungal enzymatic activity of this gut isolate. In accordance with this, such type of fungal cell wall disorientation is often considered to be an impressive biocontrol efficiency of bacterial antagonism against fungal pathogens (50). Such antifungal enzymatic capabilities of the gut isolates can help protect the host gut environment against pathogenic fungal invasion during food uptake, as well as in the digestion of the host’s chitinaceous and proteinaceous food materials.

### *In vitro* and *in silico* plant growth-promoting attributes of CMF

#### IAA production

**Fig 2:**
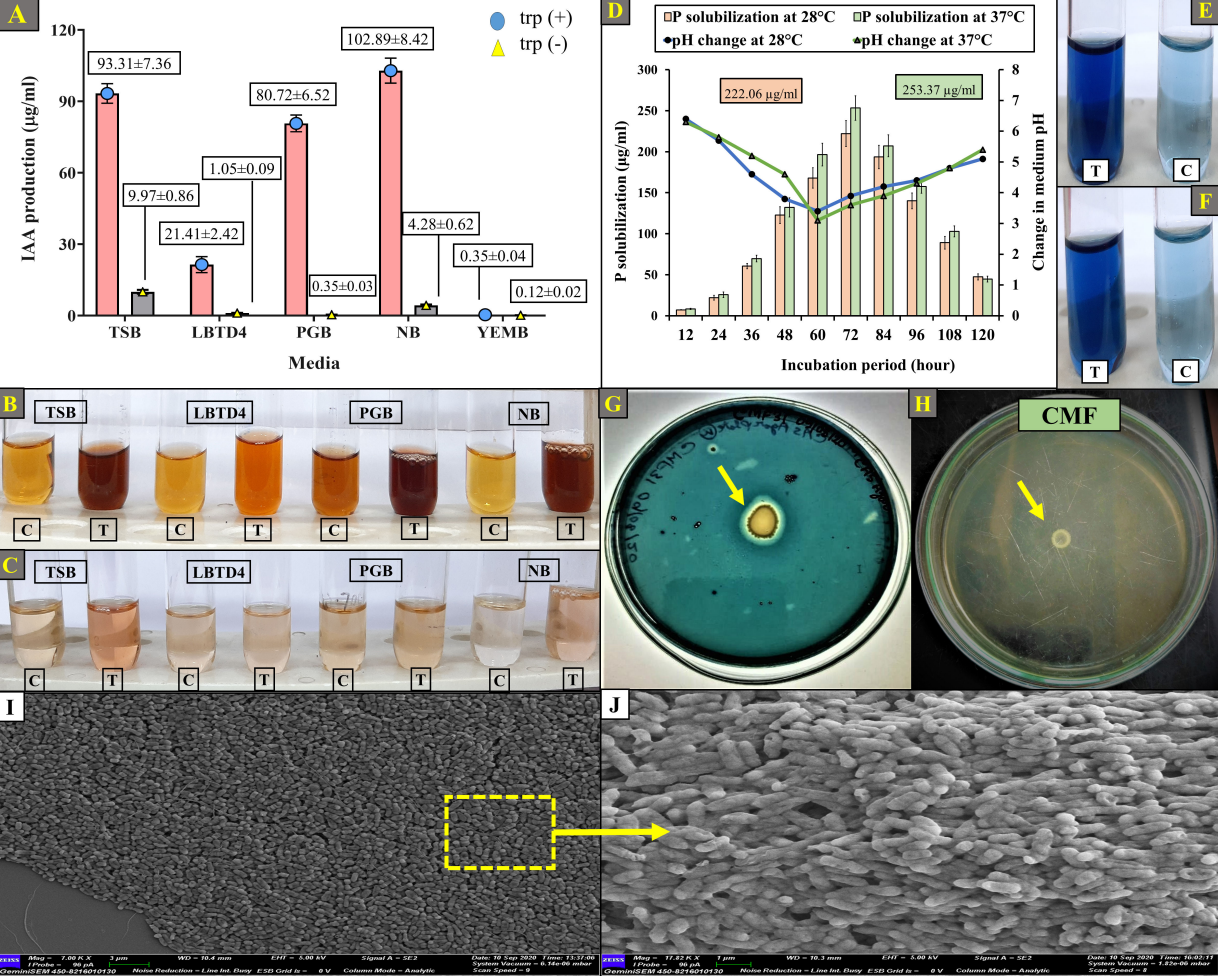
Plant growth-promoting attributes of the gut isolate *A. veronii* CMF: (**A–C**) IAA production ability of CMF, (**B**) tryptophan-supplemented media, and (**C**) tryptophan-free media. (**D–F**) P-solubilizing efficiency of CMF: (**E**) at 37°C and (**F**) at 28°C. (**G**) Siderophore production by CMF. (**H**) N_2_ fixation by CMF. (**I and J**) SEM images of biofilm-forming potential of CMF.

*In vitro* IAA production by the gut isolate CMF revealed that the highest IAA production was 102.89 ± 8.42 µg/mL in response to the L-tryptophan-supplemented NB media (Fig. 2A and B). In contrast, in the case of L-tryptophan-free culture media, CMF exhibited utmost IAA production up to 9.97 ± 0.86 µg/mL in TSB medium (Fig. 2A and C). Additionally, the insect gut isolate CMF also produces 93.31 ± 7.36, 21.41 ± 2.42, 80.72 ± 6.52, and 0.35 ± 0.04 µg/mL of IAA in tryptophan-supplemented media, i.e., TSB, LBTD4, PGB, and YEMB, respectively. However, in the same tryptophan-free media, IAA production by CMF was executed up to 1.05 ± 0.09, 0.35 ± 0.03, 4.28 ± 0.62, and 0.12 ± 0.02 µg/mL of IAA in tryptophan-free media, i.e., LBTD4, PGB, NB, and YEMB, respectively. Moreover, CMF has displayed its capability to produce IAA in both the experimented tryptophan-supplemented and tryptophan-free media, respectively (Fig. 2A through C). In this regard, IAA-producing *Aeromonas* spp. have also been documented from the gut system of the earthworm *Aporrectodea molleri*, with 78.5 µg/mL IAA production from *A. encheleia* TC22 (51), along with different *Aeromonas* spp. from diverse ecological origin (52). As an insect gut symbiont, this potential is yet to be reported. Further, IAA-producing *A. veronii* strain C7_8 was recently reported from maize rhizosphere, which is responsible for integrated PGP traits along with drought-tolerant potential (53). Engagingly, this current study corroborates insights from the CMF genome, where different L-tryptophan-dependent and -independent IAA production-related proteins—viz*.*, aminodeoxychorismate synthase, anthranilate synthase, anthranilate phosphoribosyltransferase, tryptophanase, tryptophan synthase, and indole-3-glycerol-phosphate synthase were identified (Table S6), as also documented by reports such as Wang et al. (54).

IAA production ability from the symbionts of the versatile gut systems, i.e, earthworm, insect, mice, different animals, and interestingly human gut symbionts (55–58), has also been reported. The reason behind this kind of aptitude of the gut symbionts is that gut microbiome–facilitated tryptophan catabolism appears to be one of the vital regulatory factors essential for the central nervous system or gut-brain axis in both human and animal systems (25, 59, 60). Other than the involvement of IAA-producing gut microbes in neurological disorders, it has been extensively considered for its role as aryl hydrocarbon receptor (AhR) in the management of intestinal immunity (24). In addition to that, IAA has also been anticipated to have a substantial role in bacterial signaling, colonization, and resistance to pathogenic colonization in terms of bacterial infection, which are the phenomenal criteria for gut microbial symbiosis (61).

#### Phosphate solubilization

Quantification of the phosphate solubilizing efficiency of the gut isolate revealed that the CMF is able to solubilize insoluble phosphate maximally up to 253.37 ± 12.47 and 222.06 ± 11.82 µg/mL at 37°C and 28°C, respectively, after 72 h of incubation (Fig. 2D through F). In addition to that, a prominent reduction in pH of the culture medium was also detected, where the pH of the NBRIY medium dropped from initial 7.0 to pH 3.9 and 3.6 at 37°C and 28°C, respectively, after 60 h of incubation (Fig. 2D). The reason behind the sharp decline in medium pH during the P-solubilization is that most of the proficient phosphate solubilizers decrease the medium pH by secreting organic acids, such as gluconic acid and 2-ketogluconic acid, which act as chelating agents of the cations like Al^3^+ and Ca^2^+, primarily responsible for the formation of insoluble phosphates (62, 63). Thus, a typical decrease in medium pH along with an increase in soluble phosphate production was observed over time in this study.

In comparison to the P-solubilizing efficiency of *A. veronii* CMF, a similar kind of result up to 283 ± 12 µg/mL was also reported from *Enterobacter cloacae* EAM35, a rhizospheric soil isolate, where heightened P-solubilization was achieved at reduced medium pH of 3.9 (64). However, among the *Aeromonas* sp. members, the only report of *A. encheleia* TC22, a gut isolate from earthworm *Aporrectodea molleri*, can produce soluble phosphate up to 32.1 ± 0.26 µg/mL (51). Furthermore, scientific reports show soluble phosphate production of 29.6 ± 0.15, 148.4 ± 4.26, 216.9 ± 1.2, and 215.4 ± 0.6 µg/mL after 72 h of incubation by *B. subtilis* TC34, *B. licheniformis* KX657843, *B. safensis* BSM 3, and *B. flexus* CDB2, respectively, isolated from the gut systems of *A. molleri* and *M. posthuma* (31, 39, 51). Besides these studies, several reports are also available from several phosphate solubilizers of versatile gut systems, i.e., earthworm, termite, and insects (31, 65–67). Phenomenally, the P-solubilizing ability of the gut symbionts relies on the production of alkaline phosphatase by the symbionts, which elevate the intestinal commensal bacterial growth by turning off (dephosphorylation) the luminal ATP and other luminal nucleotide triphosphates; otherwise, dysbiosis will occur and negatively affect the health and well-being of the host (26). Interestingly, this current study corroborates with the genomic characterizations of the gut isolate CMF, where different kinds of acid phosphatase (AphA), alkaline phosphatases, phosphatase, and exopolyphosphatase were found (Table S6), all of which are well-known for their phosphate solubilization potential (68).

#### Siderophore production

Evaluation of siderophore production by the gut isolates was detected by halo zone formation around the colony of the CMF (1.4 ± 0.1 cm) on the experimented CAS agar plates (Fig. 2G). It suggests that the gut symbionts can produce a moderate amount of siderophore. Further studies are needed to enhance their siderophore production capability. Among the *Aeromonas* genus, a commonly found siderophore is enterobactin, and a similar report is also available from *A. salmonicida* CBA100 (69, 70). Phenomenally, gut symbionts expressed their enterobactin-mediated iron uptake and restricted iron availability in the gut system when the pathogens, e.g., *Salmonella typhimurium*, reside within gut luminal neutrophils (71, 72). On the other hand, within the CMF genome, genomic characterization identified different siderophore production-related proteins, i.e., iron ABC transporter permease, iron-siderophore ABC transporter substrate-binding protein, enterobactin biosynthetic proteins, 2,3-dihydro-2,3-dihydroxybenzoate dehydrogenase, and Fe^3+^-hydroxamate ABC transporter permease (Table S6). Here, 2,3-dihydro-2,3-dihydroxybenzoate dehydrogenase was found to be involved in the conversion of isochorismate into 2,3-dihydroxybenzoic acid (DHBA) during the initial stages of enterobactin biosynthesis. Moreover, DHBA serves as a key precursor in the biosynthesis of many catecholate type of siderophores. On the other hand, the rest of the proteins constitute components like TonB-dependent outer membrane receptor and the ABC transporter system, which are involved in the recognition and active import of different ferric-siderophore complexes (73–75). This corroborates the siderophore production proficiencies of CMF.

Regarding the siderophore-producing gut microbial studies, several gut isolates from different gut environments, i.e., earthworms, roundworms, insects, and humans, have also been documented (67, 76, 77). In a specific way, only the report of *Aeromonas* sp. as *A. hydrophila* LC13, a gut isolate of *A. molleri*, exhibited siderophore production capability, which accounts for its PGP potential (67). Siderophore-mediated PGP activities of microbes are based on the formation of soluble Fe^3+^ complex for easy acceptance by plants, as well as competing with phytopathogens by chelating iron molecules (78). Along with their PGP attributes, siderophore-producing gut bacteria also facilitate their host systems: by (1) sequestering the iron from ingested food materials and diminishing the iron load to help overcome iron toxicosis (mainly observed in insects) (27) and (2) competing with the opportunistic pathogens and entomopathogenic fungi by chelating the iron molecules, which are required for their nutrition, metabolism, growth, and ultimately, pathogenicity (28). Additionally, siderophores are also reported to be actively involved in heavy metal bioremediation and promote plant growth under heavy metal stress conditions (73). Moreover, siderophore biosynthesis and subsequently iron-scavenging potential are not abilities equally harbored by all resident gut bacteria. From this point of view, it can be stated that siderophore production is a major criterion for the establishment of symbiosis within the host gut system.

#### N_2_ fixation

In accordance with these gut symbiotic aptitudes, the N_2_ fixation capability of the gut isolate CMF was evaluated, where CMF exhibited profuse growth (Fig. 2H) in the N_2_-free medium, reflecting its ability to utilize atmospheric nitrogen for cellular protein synthesis. Regarding the N_2_-fixing potential of the studied gut isolate, *A. encheleia* TC22 from the gut of *A. molleri* also exhibited their N_2_-fixing ability by their prolific growth in the N_2_-free medium (51). Furthermore, in a recent study, N_2_-fixing skill was also observed from termite gut symbiont *B. subtilis* (66). Similar scientific reports, apart from the *Aeromonas* sp., were also documented from the gut symbionts like *Serratia proteomaculans* 2ACDF, *Rahnella aquatilis* 6-DR, and *Pseudomonas* sp. PRGB06, isolated from *Dendroctonus rhizophagus*, *D. valens*, and *Plutella xylostella*, respectively (79). Apart from this phenotypic behavior, genomic depiction of CMF also revealed several proteins related to N_2_ fixation such as dinitrogenase iron-molybdenum cofactor biosynthesis domain-containing protein, glycosyltransferase SypJ, AAA family ATPase, NADH: ubiquinone reductase, nitrogen regulation protein NR(I), and nitrogen regulation protein NR(II), all of which are recognized for nitrogenase activity, nitrogen metabolism, and nitrogen fixation (Table S6). Such genomic-phenotypic evidence of N_2_-fixing capability of *A. caviae* A1-2 from maize rhizosphere was also reported by Babalola et al. (41).

Nitrogen, a key nutritional constituent required for the growth of all living organisms, is present in the environment but mostly exists in forms inaccessible to plants and animals. In this way, nitrogen-fixing bacteria (1) convert nitrogen (N_2_) gas into ammonia (NH_3_) for plant systems (80) and (2) recover nitrogen from uric acid, thereby preserving the nitrogen for the host beneficial activity in animal systems, especially in insects (30). In the case of plant systems, several rhizospheric microbes are responsible for N_2_ fixation, whereas in animal systems, oligonitrotrophic, wood-eating termites (*Reticulitermes flavipes*) harbor gut bacteria such as *Streptococcus* sp. and *Bacteroides termitidis* that perform this kind of N_2_ conversion. In this context, various gut microbes play an indispensable role in the recovery of nitrogen from uric acid, thus preserving the nitrogen for the host activity. The reason behind such gut symbiotic potential is that such termite diets contain only 0.03–0.15% nitrogen with a carbon-to-nitrogen ratio of 400:1, and consequently, conservation of nitrogen is essential for them (7, 30).

#### Biofilm formation

In this study, *in vitro* biofilm formation by the gut isolate CMF was detected under the SEM observation, which indicates the adhesion of bacterial cells to the solid surface. In addition to that, this bacterial strain *A. veronii* CMF also exhibited the multilayered, three-dimensional co-aggregation and tended to attach to solid surfaces (Fig. 2I and J). In this context, substantial evidence is also available from different gut systems, e.g., fish, insects, reptiles, and humans (29, 81–85). However, biofilm-forming insect gut symbionts, especially from *C. megacephala,* along with the PGP attributes, are yet to be reported. In addition to this finding, genomic insights of CMF exhibit various biofilm-forming proteins, including diguanylate cyclase, exopolysaccharide biosynthesis protein, YjbF family lipoprotein, YqcC family protein, GGDEF domain-containing protein, biofilm dispersion protein BdlA, and YhcH/YjgK/YiaL (DUF386) family protein. The potential of such proteins is mostly related to cell adhesion involved in single-species biofilm formation, exopolysaccharide biosynthesis, intracellular signal transduction, and biofilm dispersion (Table S6).

The inherent ability of microorganisms is measured by their biofilm formation, a tissue-like, multicellular, three-dimensional association of exopolysaccharide and microbes that can be connected to biotic or abiotic surfaces and tolerate several forms of stresses, such as desiccation, antimicrobial substances, the immune response of higher organisms, and the pathogenic colonization (86, 87). Inside the gut, the internal surface of the gastrointestinal tract is commonly wrapped by microbial biofilms, which play an important role in the growth and effectivity of the host organism, defend against pathogens, and maintain “gut health” (29). On the other hand, biofilm-forming rhizospheric microorganisms can elevate plant growth by alleviating stresses from salinity, heavy metals, and hazardous pollutants stress along with the N_2_ fixation and micro- and macronutrient assimilation (88–90).

### *In vivo* plant growth-promoting proficiencies of CMF

#### Plant growth promotion study of CMF in pot experiment

**Fig 3:**
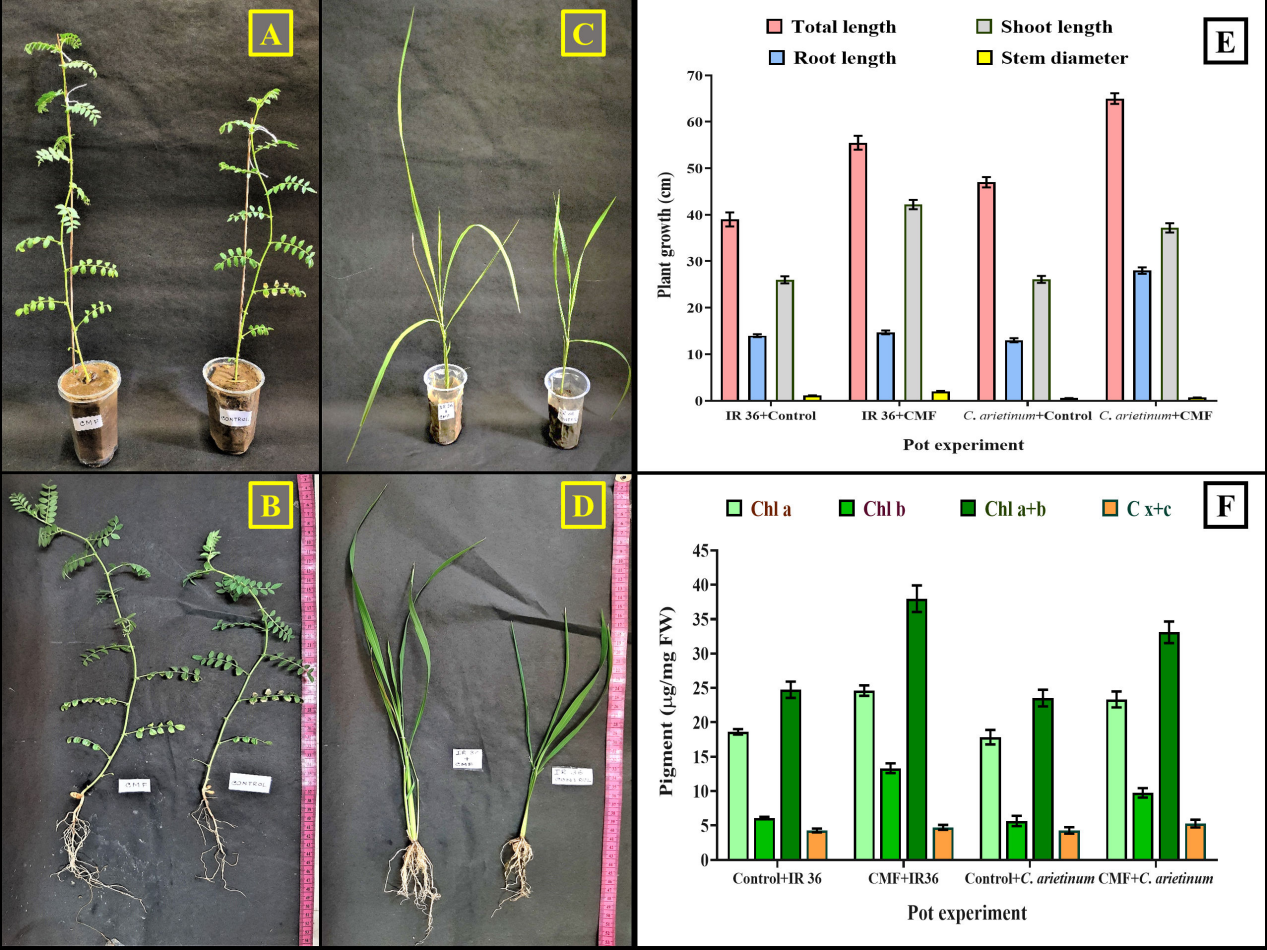
Phenotypic plant growth-promoting attributes of the gut isolate *A. veronii* CMF in pot experiment. (**A**) *C. arietinum* treated with CMF. (**B**) CMF treated whole plant of *C. arietinum.* (**C**) *O. sativa* IR36 treated with CMF. (**D**) CMF treated whole plant of *O. sativa* IR36. (**E**) Morphological promotions of CMF treated and untreated *C. arietinum* and *O. sativa* IR36. (**F**) Chlorophyll and carotenoid estimation in CMF treated and untreated *C. arietinum* and *O. sativa* IR36.

The visible effect of the plant growth promotion by the gut isolate CMF-treated model plants, i.e., *C. arietinum* L. and *O. sativa* L. IR36, in comparison to the untreated plants was evaluated in a pot-based *in vivo* experiment. At the age of 21 days, a noticeable increase in the total length (TL), root length (RL), shoot length (SL), and stem diameter (SD) of the CMF-treated model plants was observed (Fig. 3A through D). Here, CMF promoted growth in terms of TL, RL, SL, and SD of the *C. arietinum* to 65.2 ± 1.13, 28.4 ± 0.7, 37.2 ± 1.15, and 0.67 ± 0.03 cm, respectively, in comparison to CMF-untreated plants, where the same parameters were 47.2 ± 1.2, 13.2 ± 0.54, 26.1 ± 0.86, and 0.55 ± 0.02, respectively. Further, CMF-treated IR36 plants exhibited heightened growth in TL, RL, SL, and SD, up to 55.5 ± 1.58, 14.7 ± 0.44, 42.2 ± 1.4, and 2.0 ± 0.1 cm, respectively. In contrast, the control sets of plants recorded 39.2 ± 1.62, 14.0 ± 0.4, 26.3 ± 0.94, and 1.10 ± 0.05 cm for the same parameters, respectively (Fig. 3E). After the heightened morphological evidences, another aspect of plant growth promotion, namely, positive changes in chlorophyll (Chl a, Chl b, and Chl a + b) and total carotenoid (C x + c) contents, was evaluated in CMF-treated model plants. Here, *C. arietinum* L. plants exhibited increased levels of Chl a, Chl b, Chl a + b, and C x + c contents, up to 23.34 ± 1.15, 9.75 ± 0.69, 33.10 ± 1.57, and 5.29 ± .056 µg/mg of fresh weight of specific plant parts (FW), respectively, when treated with gut isolate CMF, compared to the control plants, which showed 17.85 ± 1.71, 5.68 ± 0.76, 23.53 ± 1.61, and 4.29 ± 0.46 µg/mg FW, respectively. Similarly, *O. sativa* L. IR36 plants showed their elevated Chl a, Chl b, Chl a + b, and C x + c contents up to 24.62 ± 0.76, 13.34 ± 0.77, 37.96 ± 1.92, and 4.74 ± 0.35 µg/mg FW, respectively, compared to CMF-untreated plants, which had 18.62 ± 0.86, 6.12 ± 0.17, 24.75 ± 1.19, and 4.28 ± 0.27 µg/mg FW, respectively (Fig. 3F).

In the search for phenotypic or morphological PGP attributes, few documented studies are available regarding the insect (*Helicoverpa zea*) and earthworm (*A. molleri* and *M. posthuma*) gut isolates, where the model plants mainly included *Brassica napus*, *Capsicum annuum*, *Solanum lycopersicum*, *Zea mays*, *Vigna radiata,* and *V. radiata* (31, 65, 67, 82, 91, 92). However, studies about the plant growth promotional attributes of the insect gut bacteria on *C. arietinum* and *O. sativa* are yet to be reported. Moreover, gut bacteria-mediated PGP attributes in terms of enhanced chlorophyll and carotenoid contents in any agronomic plants have not been evaluated so far.

#### Plant root colonization potential of CMF

**Fig 4:**
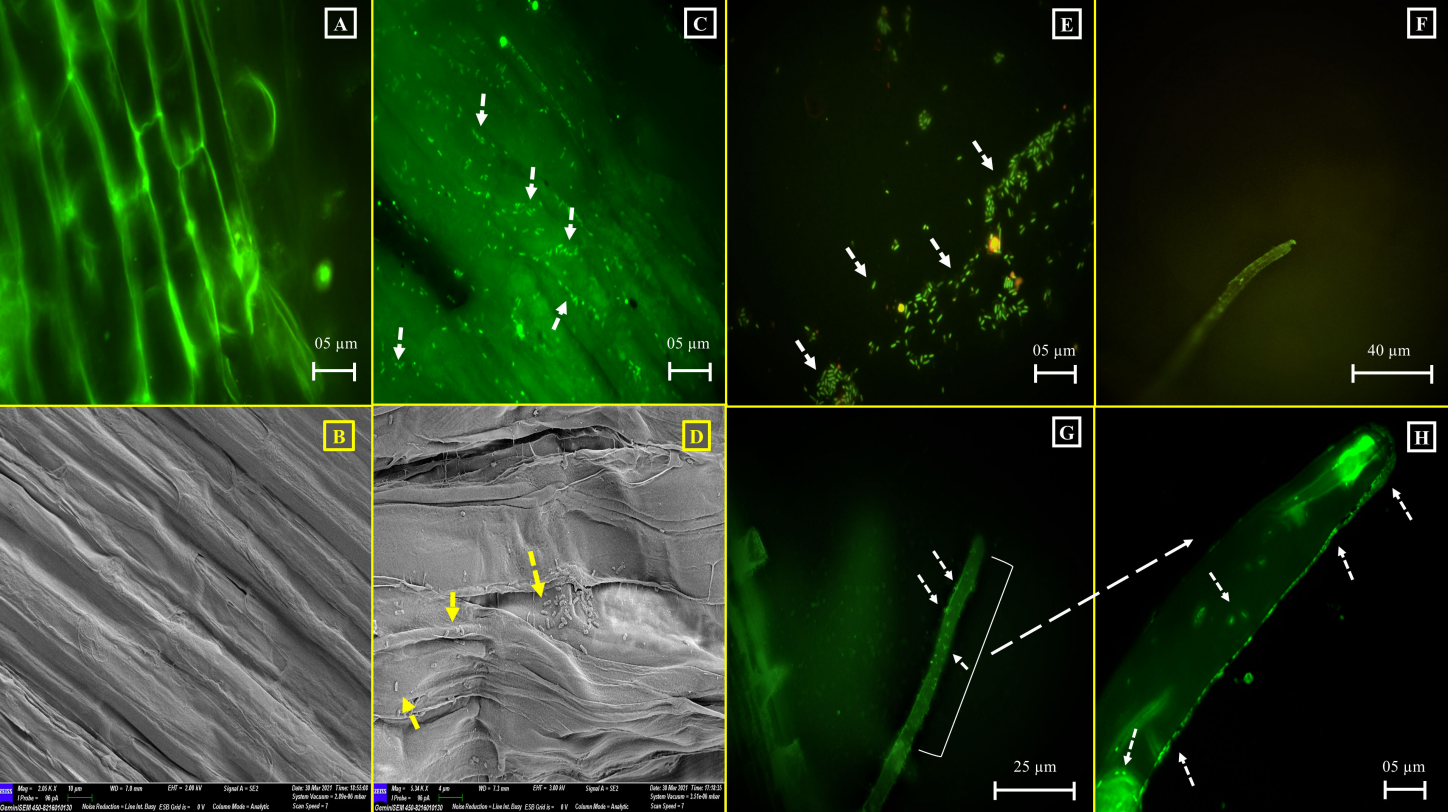
Plant root colonization potential of the gut isolate *A. veronii* CMF in *C. arietinum.* (**A and B**) Root surface of *C. arietinum* without CMF inoculation: (**A**) FM study (**B**) SEM study. (**C and D**) CMF-colonized root surface of *C. arietinum*: (**C**) FM study and (**D**) SEM study. (**E**) FM image showing CMF colony. (**F**) Single root hair of *C. arietinum* without CMF inoculation. (**G and H**) FM study of CMF-colonized single root hair of *C. arietinum*.

**Fig 5:**
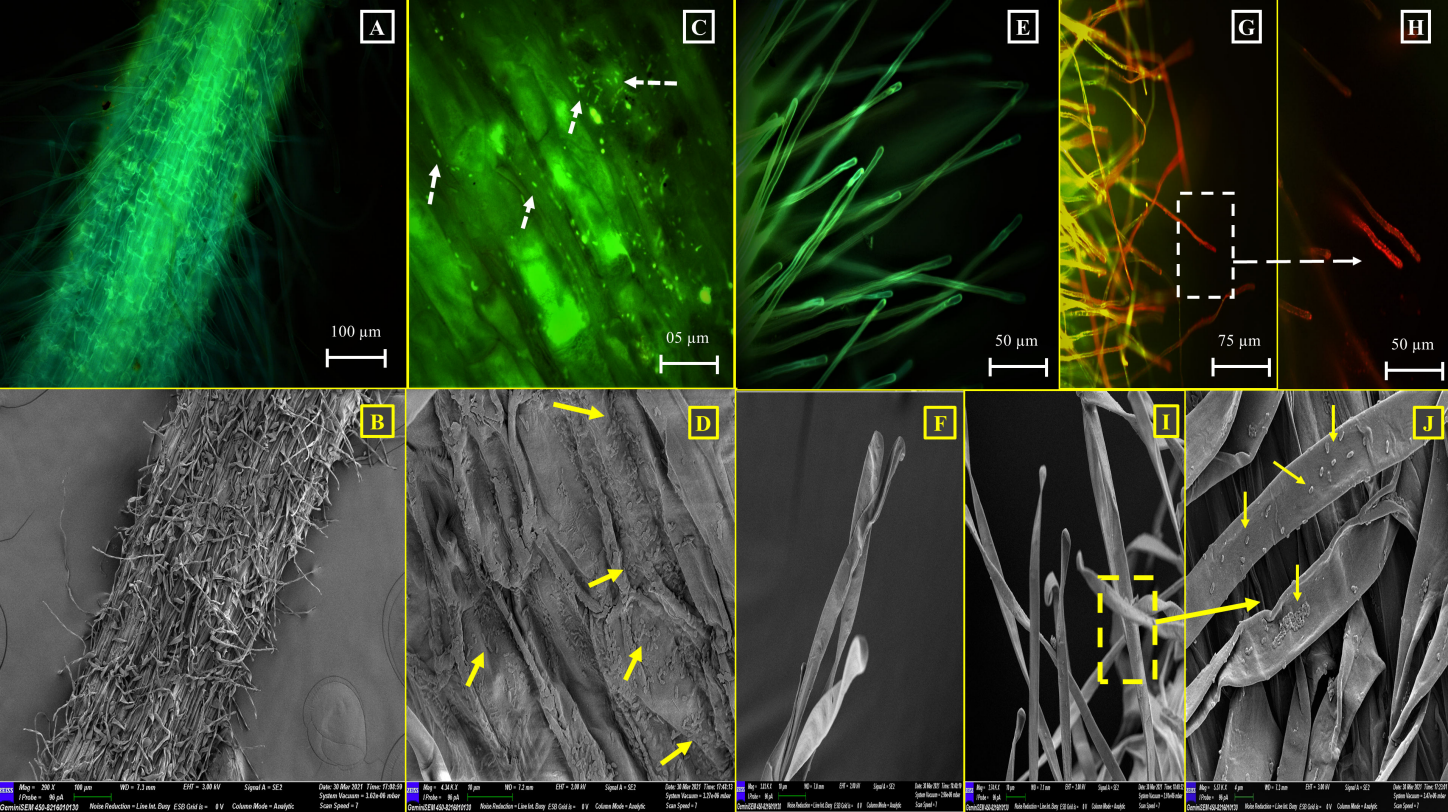
Plant root colonization potential of the gut isolate *A. veronii* CMF in *O. sativa* IR36. (**A and B**) Root surface of *O. sativa* IR36 without CMF inoculation: (**A**) FM study and (**B**) SEM study. (**C and D**) CMF-colonized root surface of *O. sativa* IR36: (**C**) FM study and (**D**) SEM study. (**E and F**) Single root hair of *O. sativa IR36* without CMF inoculation: (**E**) FM study and (**F**) SEM study. (**G and H**) CMF-colonized single root hair of *O. sativa* IR36 (FM study). (**I and J**) CMF-colonized single root hair of *O. sativa* IR36 (SEM study).

The optimum consequence of plant-PGP bacterial interaction, in terms of augmented plant growth, depends on the degree of successful colonization of associating bacteria (93). In this context, the potential of CMF to colonize in the *C. arietinum* and *O. sativa* IR36 plant roots was evaluated. The capability of root colonization was checked 15 days after inoculation of the experimented plantlets with the tested gut isolate CMF. In this endeavor, CMF successfully colonized the root surfaces of the tested model plants , with accounts ranging from 1.08 × 10^3^ to 1.1 × 10^3^ CFU/gm of the root, respectively. However, no bacterial colonization was observed on root surfaces or root hairs of *C. arietinum* (Fig. 4A and B, F) and *O. sativa* IR36 (Fig. 5A and B, E and F) in the uninoculated control sets. Furthermore, fluorescence microscopic (FM) and SEM observations evidently revealed that an ample number of gut bacterial cells were associated both in the primary roots and root hairs of *C. arietinum* (Fig. 4C and D, G and H) and *O. sativa* IR36 (Fig. 5C and D, G through J). Such colonization potential and subsequent relationship development by gut symbionts often starts as a pathogenic one but then evolves to a beneficiary transaction and becomes mutualistic. However, once a pathogen transitions into a mutualist, it is typically unable to regain its pathogenic traits (94). Thus, the root-colonizing competency of the gut isolate *A. veronii* CMF is established both in monocotyledonous and dicotyledonous plants. In this way, the insect gut isolate CMF fulfills the foremost necessary PGP aptitude, root colonization, in a noteworthy manner. A similar visualization-based plant root colonization study was also reported by Singh and Jha (95), where rhizospheric isolate *S. marcescens* CDP-13 was found to colonize the roots of *Triticum aestivum* L. as part of a beneficial plant-microbe interaction. However, successful *in vivo* plant root colonization mediated by gut bacteria has not yet been exclusively reported from insect gut symbionts.

Genomic insights of CMF reveal that several OmpA family proteins and flagellins (flaA and flaB) are present, which are responsible for porin activity and flagellum-dependent swarming motility (Table S6). Porin is commonly reported as root adhesins, produced by plant root-colonizing bacteria, and reports are also available regarding such studies (96). Further, flagellin is also important for bacterial motility, a key activity required for host colonization by both pathogens and symbionts (97). During this root colonization process, bacteria alter their physiological structures and release adhesive compounds throughout the attachment process. For instance, in order to overcome electrostatic repulsion, bacteria utilize flagellar movement and secrete porins (98, 99).

### *In vitro* and *in silico* bioremediational attributes of CMF

#### Heavy metal resistant/tolerant potential of the gut isolate CMF

To understand the HM (As^3+^, Cd^2+^, Cr^6+^, Cu^2+^, Pb^2+^, and Hg^2+^) resistance or tolerance capability of the CMF, minimum inhibitory concentrations (MICs) of these HMs were determined. At first, a primary screening of MICs of the CMF was performed with tested HMs at 300, 500, and 1,000 µg/mL concentrations. In this assay, it is clearly visible that CMF can easily resist/tolerate all the tested HMs up to 1,000 µg/mL except mercury (Fig. S18). Therefore, extensive studies were made to determine the particular MIC of the gut isolates against the tested HMs. The current analysis revealed that the insect gut isolate CMF can resist/tolerate up to 1250 ± 25.0, 1200 ± 20.0, 1250 ± 25.0, 1200 ± 20.0, 1200 ± 20.0, and 10 ± 1.0 µg/mL of arsenic, cadmium, chromium, copper, lead, and mercury, respectively (Fig. S19). It is relevant to mention that a similar study has been reported by Biswas et al. (31), where *B. megaterium* PSB1, *S. haemolyticus* PSB2, and *B. licheniformis* PSB3 from gut system of *M. posthuma* can tolerate up to 600 and 200 µg/mL of copper and zinc, respectively. In contrast, such bioremediating proficiencies from insect gut symbionts are still to be reported and hold ample scope for exploration.

#### Heavy metal removal efficiency of the gut isolate CMF

**Fig 6:**
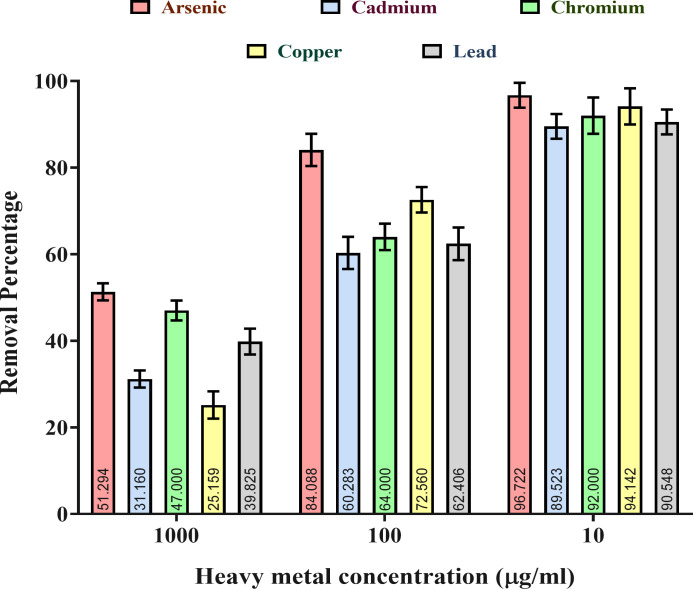
Heavy metal removal potential of the gut isolate *A. veronii* CMF.

In accordance with the HM resistant or tolerant characteristic of the gut isolate *A. veronii* CMF, the highest HM removal proficiency of the CMF was also ascertained through AAS study. In this endeavor, CMF displays its utmost removal proficiencies consistently over arsenic, copper, and chromium, followed by lead and cadmium (Fig. 6). In this way, CMF exhibited arsenic removal efficiency of up to 96.72±2.86%, 84.08±3.16%, and 51.29±2.74% at concentrations of 10, 100, and 1,000 µg/mL, respectively. Further, CMF showed copper removal capability of up to 94.14±4.18%, 72.56±3.11%, and 25.15±3.17% when treated with 10, 100, and 1,000 µg/mL concentrations, respectively. Thereafter, chromium removal efficacy of CMF was 92.00±4.76%, 64.00±3.68%, and 47.00±2.84% upon exposure to 10, 100, and 1,000 µg/mL concentrations of the HM, respectively. These findings strongly corroborate the genomic characterization of the strain CMF where different types of enzyme coding genes and transporter genes are present (Table S7). It can be assumed that these are responsible for the removal potential of such HMs. Moreover, further research study on the bioaccumulation of HMs is aimed at the best three HM removal efficiencies, i.e., arsenic, copper, and chromium, by this gut isolate, CMF. Regarding such HM removal potential, any kind of insect gut symbiont has not been reported; gut isolates with such novel characteristics can be harnessed for multidimensional sustainable approaches.

#### Heavy metal accumulation potential of the gut isolate CMF

**Fig 7:**
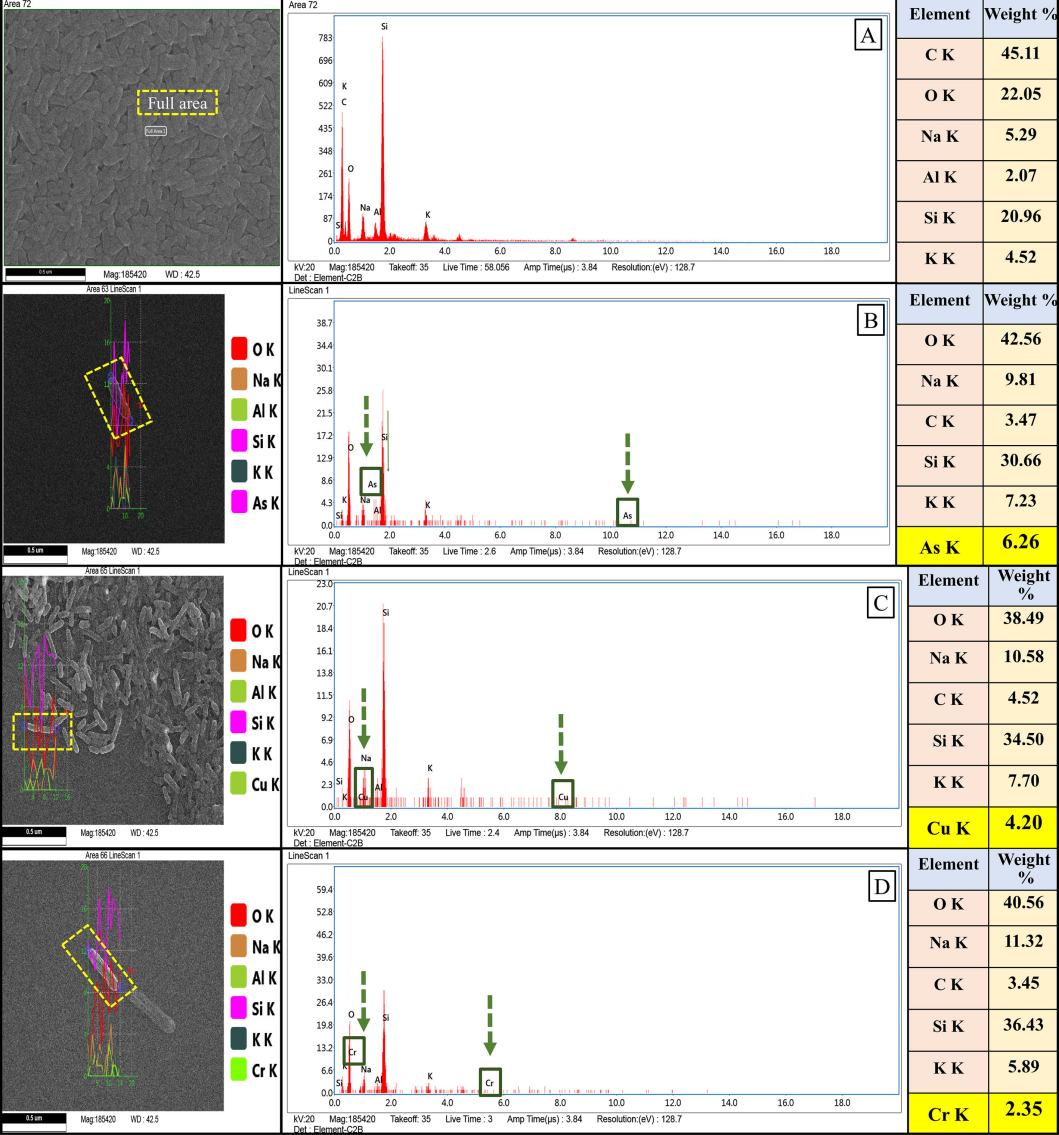
SEM-EDXS analysis of intracellular heavy metal accumulation in individual *A. veronii* CMF cell. (**A**) HM untreated CMF cells with no HM accumulation. (**B**) Intracellular arsenic accumulation potential of individual CMF cell. (**C**) Intracellular copper accumulation potential of individual CMF cell. (**D**) Intracellular chromium accumulation potential of individual CMF cell.

Bioaccumulation is an instinctive biological phenomenon where microbes utilize their biomolecules along with the cellular energy-dependent pumping and transporter systems to assimilate and sequester metal ions in the intracellular space and employ them for cellular processes (100–102). In this study, SEM-EDXS–derived analysis shows visible peaks in arsenic-, copper-, and chromium-treated cells compared to the absence of HM peaks in untreated bacterial cells, which defines the bioaccumulation properties of the CMF (Fig. 7). Here, *A. veronii* CMF exhibited 6.26% of arsenic (Fig. 7B), 4.20% of copper (Fig. 7C), and 2.35% of chromium (Fig. 7D) accumulation relative to the HM-free control set (Fig. 7A). Such experimented bioaccumulation properties of this gut isolate were further validated by genetic characterization, which identified energy-dependent pumping and transporter systems (Table S7). It can be assumed that these features are responsible for their host beneficial activity against HM toxicity. In this aspect, reports are available regarding insect gut bacteria (103). Although rhizospheric PGP microbes-mediated HM bioaccumulation is measured as one of the most operative techniques to reduce phytotoxic effects, several HM-resistant gut microorganisms, such as *V. splendidus*, *Shewanella pasifica*, *Bacteroides plebeius*, *Faecalibacterium prausnitzii*, and *Lactobacillus reuteri*, have also been reported from diverse gut systems (e.g., sea squirt, earthworm, insect, bird, and human). These microbes help restrict HM toxicity for their respective hosts (103–108).

With connection to the HM degradation, versatile HM detoxifying genes, proteins, chaperones, and transport systems were also detected from the genome of the CMF (Table S7). In case of arsenic removal capability, ArsC (glutaredoxin) family arsenate reductase, Acr3 family arsenite efflux transporter, arsenical pump-driving ATPase, and arsenite efflux transporter metallochaperone ArsD were documented. These are cumulatively responsible for the conversion of arsenate to arsenite and further extrusion of arsenite from the cells, along with the efflux transporters and arsenical pump-driving ATPase. Genomic evidence related to the presence of ArsC and ArsD in *A. allosaccharophila* was also reported from the branchiae, scales, and cloaca of *Mugil brasiliensis* and *Caranx latus* (109). Moreover, the existence of cadmium-translocating P-type ATPase, divalent metal cation transporter MntH, divalent metal cation transporter FieF, cation diffusion facilitator family transporter, and zinc/cadmium/mercury/lead-transporting ATPase in the CMF genome also reflects the cadmium removal ability of CMF. Such proteins and transporter systems are commonly known for removing/translocating cadmium ions across biological membranes of prokaryotes/plants and are involved in metal tolerance/resistance through ion efflux. A similar cadmium-translocating P-type ATPase was also reported from a spoiled milk-borne *A. hydrophila* (110). Furthermore, a chromate efflux transporter was detected in the CMF genome, which is generally responsible for efflux of chromate ions from the cytoplasm and resistance against chromate (111). In addition, the CMF genome is well organized with multiple copper detoxifying proteins, transporters, and chaperones, including copper homeostasis protein CutC, divalent-cation tolerance protein CutA, thioredoxin domain-containing protein, efflux RND transporter permease subunit, and copper chaperone PCu(A)C. These machineries are involved in upholding copper homeostasis, disulfide oxidoreductase activity, transmembrane transporter activity, and reducing high copper chelation in the cytoplasm at the cellular level, which reflects the copper resistance, tolerance, removal, and sensing potential, as reported in different PGP bacteria (112, 113). In this way, genome annotation of CMF also identified zinc/cadmium/mercury/lead-transporting ATPase, indicating its potential for lead resistance and removal. This P-type cation transport ATPase is predicted to be responsible for the active lead bioremediation process and serves as a point of reference for the multiple HM resistance potential of bacteria (114). Lastly, for mercury remediation, pyridine nucleotide-disulfide oxidoreductase was detected in the CMF genome, which is commonly known for its mercury (II) reductase (NADP+) activity. This oxidoreductase is very similar to the glutathione reductase (GR) and is predicted to be a part of the *mer* operon, which is known to induce the alteration of inorganic mercury [Hg(II)] and methylmercury (MeHg) to elemental mercury [Hg(0)] (115). Though versatile reports (genomic insights) are available regarding the HM remediation proficiency from different *Aeromonas* spp., it has yet to be reported as an insect gut symbiont. In this regard, reports are available about field implications of gut symbionts (*L. acidophilus*, *L. rhamnosus,* and *E. coli*) in soil HM bioaccessibility, including their effectiveness and persistence in the soil systems, which is completely different from their original niche (116). Moreover, experimental evidence also exists where lactic acid bacteria and versatile proteobacterial genera from insect gut systems were utilized for the reclamation of mining soils and HM-contaminated soils, respectively (103, 117–120). Gut bacteria precisely bind and accumulate toxic HMs and assist in detoxifying HMs from the host system, thereby reducing exposure (121). Their mechanism for binding HMs mainly depends on the peptidoglycan and teichoic acid ion-exchange responses (122), nucleation reactions leading to precipitation (123), complex formation via ligands (124), and chelation through siderophores (125). Compared to gut bacterial cellular mechanisms, soil bacteria also possess similar HM-remediating molecular attributes (126, 127). Therefore, it can be stated that the gut bacteria are also able to degrade HMs under soil environmental conditions. In this study, the strain CMF exhibited bioaccumulation potential, which implies its strong biosorption potential as well, indicating its effectiveness in metal-contaminated soil ecosystems.

### Conclusion

The *in silico* analysis of the gut symbiont’s genome, along with validation analysis through *in vitro* and *in vivo* studies, revealed that selected isolate *A. veronii* CMF possesses antifungal enzymatic (chitinase, protease, and β-glucanase) potential, which are also reflected by its excellent biocontrol activities against several plant and animal pathogenic fungi. The biocontrol index clearly depicts that the utmost antifungal activity of the CMF was observed against phytopathogens such as *A. alternata*, *A. niger*, and *F. solani*, respectively. Similarly, the maximum biocontrolling ability against animal pathogenic fungi by these gut isolates CMF was observed against *C. albicans* and *C. tropicalis*, respectively. In that way, gut symbiont CMF also carried out PGP traits, i.e., IAA and siderophore production, P-solubilization, N_2_ fixation, biofilm formation, and root colonization in *in vitro* conditions, which were corroborated with the genomic characterization. In continuation with the *in vitro* exploration of PGP features, promising *in vivo* root colonization potential among the *C. arietinum* and *O. sativa* IR36 by CMF was also documented in this study, strongly supporting its potential as a promising PGP gut bacterium. Additionally, significant phenotypic improvements were observed in both monocotyledonous and dicotyledonous model plants during *in vivo* pot experiments, endorsing this gut isolate as a member of the PGP bacterial category and thereby completing the spectrum of its PGP aptitudes. Thus, such biocontrol and PGP attributes of CMF, as reflected in these *in vitro* and *in vivo* studies along with the corroboration of *in silico* analysis, are actually gut symbiotic characteristics commonly performed by the gut residential microbes during their gut-symbiotic relationship. Besides its biocontrol and PGP potential, CMF also exhibited bioremediational attributes in terms of significant resistance to, removal of, and accumulation of HM (As^3+^, Cd^2+^, Cr^6+^, Cu^2+^, Pb^2+^, and Hg^2+^). Maximum HM removal and intracellular bioaccumulation capabilities were observed for arsenic, copper, and chromium among the other tested HMs, and these efficacies were also endorsed by the *in silico* genetic analysis of the gut isolate.

Therefore, this study concludes with the interesting observation that such integrated antifungal, PGP, and bioremediational attributes from any gut symbiotic microbial resources have yet to be reported. Hence, it can be stated that the isolate *A. veronii* CMF from *C. megacephala* gut system can be harnessed as a novel biocontrolling, biofertilizing, and bioremediating agent in the arena of plant biotic and abiotic stress management, potentially replacing the conventional fungicides and chemical fertilizers in the agricultural fields. Again, in this context, CMF can be explored as an eco-sustainable tool for reducing the agronomic losses caused by the phytopathogens, as well as for diminishing HMs accumulation in agronomic plant systems due to the severe environmental pollution.

## MATERIALS AND METHODS

### Isolation and identification of *C*. *megacephala* and gut bacteria CMF

To identify the selected insect, adult and healthy specimens were deposited and identified from the Zoological Survey of India, Kolkata. Isolation and identification of the gut bacteria were described in our previous study (18).

#### Pathogenicity test of the gut isolate CMF

The pathogenic potential of the isolate CMF was assessed using hemolytic activity on blood agar (Himedia, India) consisting of 0.5% defibrinated sheep blood (Sigma-Aldrich, USA), and DNase activity was determined using DNase agar (Himedia, India) supplemented with Calf thymus DNA (Sigma-Aldrich, USA) (128, 129). Pathogenic strain *B. subtilis* MTCC 121 was used as a positive control for these experiments.

### Antifungal enzyme production potential of CMF

#### Determination of chitinase production by CMF

Chitinase production capability of the isolate CMF was determined both qualitatively and quantitatively. In qualitative estimation, strain CMF was grown on chitinase-producing agar media, and its potential was measured according to Kuddus and Ahmed (130) (Table S2). Quantitatively, chitinase production by CMF was ascertained and expressed in U/mL, following the protocol of Song et al. (131). One unit (U) of chitinase production was determined as the amount of enzyme that produced 1 mM of N-acetylglucosamine (GlcNAc) per hour at 37°C. GlcNAc was used as a standard. The control set of the reaction mixture was prepared with an equal volume of sterilized culture medium instead of cell-free supernatant as the source of chitinase. Other components were followed according to the protocol. Experiments were carried out in triplicates.

#### Ascertainment of protease production by CMF

Protease production potential of the isolate CMF was examined both qualitatively and quantitatively. In qualitative estimation, strain CMF was grown on protease-producing peptone-gelatin agar media, and its capability was measured as per the protocol of Jacob and Gerstein (132) (Table S2). Protease production by CMF was quantitatively determined and expressed in U/ml, according to the protocol of Cupp-Enyard (133). One unit (U) of protease production was measured as the amount of enzyme that liberated 1 µM of tyrosine equivalents from casein at 37°C. Tyrosine was used as a standard. The control set of the reaction mixture was prepared with an equal volume of sterilized culture medium instead of cell-free supernatant as the source of protease. Other components were followed according to the protocol. Experiments were done in triplicates.

#### Detection of β-glucanase production by CMF

β-glucanase production ability of the isolate CMF was examined both qualitatively and quantitatively. In qualitative estimation, strain CMF was grown on laminarin agar media, and its ability was measured according to the Wu et al. (134) (Table S2). Quantitative estimation of β-glucanase production by CMF was ascertained and expressed in U/mL, according to the protocol of Ghose (135). One unit (U) of β−1,3-1,4-glucanase activity was accounted as the amount of enzyme that released 1 mM of glucose per min at 37°C. Glucose was used as a standard. The control set of the reaction mixture was prepared with equal volume of sterilized culture medium instead of cell-free supernatant as the source of β-glucanase. Other components were followed according to the protocol. Experiments were done in triplicates.

#### Determination of the nature of antifungal metabolites

Antifungal characteristics of the strain CMF were tested by the agar well diffusion method (45). To execute this experiment, 100 µL of *Alternaria alternata* VBAV007 conidial suspension (2.1 × 10^4^ conidia/mL) was sprayed on malt extract (ME) agar plate, and then, wells were made in the agar plate using a sterilized cork borer. Cell-free supernatant (CFS) of strain CMF was collected after 48 h of culture growth in Luria-Bertani broth. CFS was obtained from centrifugation at 10,000 rpm for 15 min, followed by membrane filtration (0.22 µm cellulose acetate membrane). 50 µL of CFS of CMF were added to one of the wells. The thermostable nature of the antifungal metabolites produced by the gut isolate *A. veronii* CMF was investigated by keeping the CFS in a boiling water bath for 15 min. In addition, to check the proteinaceous nature of the antifungal principles, CFS was treated with proteinase K (1 mg/mL) (HiMedia, India) for 2 h at 37°C (136). The antifungal potential of both heat-killed and proteinase K-treated CFS was studied against the pathogen *A. alternata* VBAV007 by using the agar well diffusion method (45). Wells with only sterilized Luria-Bertani broth medium (non-inoculated with CMF) were considered as the control. After loading all the wells with CFS, heat-killed CFS, CFS with proteinase K, and sterilized LB (control), the plate was incubated for 72 h at 28°C for observation.

### *In silico* characterization of chitinases from CMF

Computational characterizations of the antifungal enzymes from the genome of *A. veronii* CMF were studied. Protein-coding genes of the chitinases were extracted from the whole-genome sequences of *A. veronii* CMF (described in 18). Here, in case of physicochemical characterization, the enzymatic features considered were amino acid length (AAL), molecular weight (MW), isoelectric point (pI), extinction coefficient (EC), instability index (II), aliphatic index (AI), grand average of hydropathicity (GRAVY) value, Tm index, and Tm value. On the other hand, the selected properties for secondary structure prediction were percentages of α-helix, β-sheet, and β-turn. In addition, homology protein modeling was performed for every enzyme of interest, and quaternary structures, QMEAN value, Z-score, Ramachandran plot, and ERRAT score (overall quality factor) were evaluated.

#### Physicochemical characterization of the antifungal enzymes

To perform the physicochemical characterization of the chitinases from the gut isolates *A. veronii* CMF genome, ExPASy-ProtParam tool (https://www.expasy.org/resources/protparam) was used in this study. In addition to that, to evaluate the Tm index and Tm value of the enzymes, Tm index programmer (https://www.sciencedirect.com/science/article/pii/S1476927109001091) was also carried out (137).

#### Prediction of secondary structural conformation of the antifungal enzymes

Prediction of secondary structures of the antifungal proteins like chitinases from *A. veronii* CMF genome was performed by CFSSP web-based server (http://www.biogem.org/tool/chou-fasman/) (138). In this study, percentages of α-helix, β-sheet, and β-turn of each desired enzyme were evaluated.

#### Homology protein modeling, structural assessment, validation, and submission

Homology 3D protein modeling of antifungal enzymes, i.e., chitinase from *A. veronii* CMF, was performed using Swiss model workspace by choosing a suitable and best-matched template (139). Assessment of the predicted protein models was performed in both QMEAN (Qualitative Model Energy Analysis) (47, 140) and SAVES server (Structure Analysis and Verification Server) (http://services.mbi.ucla.edu/SAVES/), including Ramachandran plot analysis. On the basis of the evaluation report, the best-built model was finally submitted to Protein Model Database (PMDB) (https://academic.oup.com/nar/article/34/suppl_1/D306/1133443), and the PMDB ID was obtained.

#### Phylogenetic analysis

To unravel the evolutionary relationship among the antifungal enzymes of CMF genome (MXV30556.1, MXV29181.1, MXV28648.1, MXV28646.1, MXV31165.1, MXV29488.1, and MXV28820.1), a phylogenetic tree was constructed using MEGA X (141). The evolutionary history was inferred using the Neighbor-Joining method (142) with 1,000 bootstrap replicates. The evolutionary distances were computed using the Dayhoff matrix-based method (143).

#### Functional analysis

STRING (Search Tool for the Retrieval of Interacting Genes/Proteins) database (https://string-db.org/ Version 12.0) has been used to predict functional interacting partners of known proteins (34). A functional protein association network was revealed for all antifungal enzymes of the CMF genome (MXV30556.1, MXV29181.1, MXV28648.1, MXV28646.1, MXV31165.1, and MXV29488.1) except MXV28820.1 (as no identical proteins were available in the STRING database related to MXV28820.1).

### Biocontrol potential of CMF against fungal pathogens

To evaluate the biocontrol efficiencies of the studied gut isolate, several kinds of antifungal investigations were demonstrated, including dual culture overlay assay, biocontrol index, fungal cell-wall lytic enzyme production assay, and field emission scanning electron microscopic (FESEM) analysis.

#### Dual culture overlay assay

*In vitro* antifungal activity of *A. veronii* CMF was performed against different plant pathogenic as well as animal pathogenic fungal strains following the protocol of dual culture overlay assay (144). The tested phytopathogens are responsible for versatile plant diseases, such as leaf spot disease of *Aloe vera*, wilt disease of pea, black root rot disease of chickpea, blight of banana, leaf spots, scab, post-harvest rots, fruit rot of jackfruit, black rot of onion and garlic, orange spoilage, and post-harvest disease in apple (Table S8). All the laboratory-isolated strains were identified by 28S rDNA sequence homologies (145). Here, bacterial culture of *A. veronii* CMF was grown on Luria-Bertani (LB) broth for overnight and streaked on the nutrient agar plates (pH 7.0), followed by 24 h of incubation at 37°C. Further, these plates were overlaid with malt extract agar (0.7% agar) containing pathogenic fungal suspension (10^3^–10^4^ spores or conidia/mL) and incubated at 28°C for 72 h (1). Thereafter, the zone of inhibition of each pathogenic fungal strain against the CMF was documented.

#### Biocontrol index

To distinguish the antifungal potential of the experimented gut isolate CMF against different kinds of fungal pathogens, a biocontrol index was prepared from the zone of inhibition (mm) and bacterial colony diameter (mm) formed during the dual culture overlay assay.


(1) $$Biocontrol\;index=\frac{Zone\;of\;inhibition}{Colony\;diameter\;of\;the\;bacterial\;isolates}$$

#### SEM analysis

In order to decipher the antifungal activity of the gut isolate CMF against the pathogenic fungal strains, mycelial morphologies of the experimented pathogens were documented and analyzed through SEM study. Fungal mycelia of the tested pathogenic strains were obtained from the inhibition zone (toward bacterial colony) formed on dual culture overlay plates. Next, pre-fixation (for 30 min) of these affected mycelial structures was carried out using 2% glutaraldehyde in 20 mM PBS-1X (pH 6.5) with 5% dimethyl sulfoxide (DMSO) (vol/vol). Further, post-fixation (for 30 min) was also executed using 1% osmium tetraoxide in 50 mM PBS-1X (pH 6.5) (vol/vol). Subsequently, fixed mycelia were dehydrated with ethanol gradients (10–100% at 10% interval for 01 min each gradation). Samples were subsequently mounted in the gold grid for 105 s (Quorum, SC76220, Sputter coater) and observed under SEM (GeminiSEM-450, Zeiss, UK). A control set was also prepared following SEM preparation method using only fresh fungal mycelia grown on ME agar plates (146).

### Plant growth-promoting attributes of CMF

To evaluate the PGP potential of the studied gut isolate CMF, several tests were carried out, including indole acetic acid (IAA) production, phosphate solubilization, biofilm formation, siderophore production, nitrogen fixation, pot experiments, chlorophyll and carotenoid estimation of the studied plants, and plant root colonization capability.

#### IAA production

*In vitro* IAA production of the gut isolate *A. veronii* CMF was estimated following the protocol of Gordon and Weber (147). The gut symbiont CMF was grown in different liquid culture media, including nutrient broth (NB), yeast extract mannitol broth (YEMB), Luria-Bertani tryptone broth (LBTD4), and peptone gelatin broth (PGB) supplemented with and without l-tryptophan (1 mg/mL) at 37°C for 72 h. After incubation, cell-free supernatants (CFS) were collected by centrifugation at 8,000 rpm for 15 min. Then, the CFS was permitted to react with 4 mL of Salkowski reagent for 20 min in the dark condition at room temperature. Thereafter, optical density (OD) of reaction mixtures was taken at 535 nm (Shimadzu 1700), and the quantity of IAA produced was calculated by comparing with the standard curve (0–200 µg/mL) prepared using pure IAA (Sigma-Aldrich).

#### Phosphate solubilization

To quantify the phosphate solubilization efficiency of the gut isolate *A. veronii* CMF, an experiment was performed according to Chen et al. (148). CMF (1% bacterial culture with OD = 0.5 at 600 nm) was inoculated to 25 mL of NBRIY medium (devoid of yeast extract and pH = 7.0) (149), where tricalcium phosphate acted as a sole phosphate source, and was incubated for 120 h at dual temperature, i.e., 28°C and 37°C, under shaking conditions at 120 rpm. After incubation, CFS was obtained by centrifugation at 8,000 rpm for 15 min. Now, 1 mL of CFS was added to 4 mL of Chen reagent and incubated for 1 h at 28°C and 37°C. Further, the OD of the reaction mixtures was measured at 619 nm (Shimadzu 1700), and the quantity of soluble phosphate produced was calculated by comparing with the standard curve (up to 500 µg/mL) prepared with dipotassium hydrogen phosphate (K_2_HPO_4_) standard (Sigma-Aldrich). Phosphate solubilizing proficiency was checked at 12-hour intervals after inoculation of the gut isolate. Additionally, at every 12 h of interval, the pH of media was also monitored.

#### Biofilm formation

The biofilm-forming capability of the gut isolate *A. veronii* CMF was studied using SEM. Experimented strains were grown in the liquid culture medium, i.e., LB and chitin-supplemented broth (130), under static conditions for 72 h at 37°C, where small pieces of glass chips (1 × 1 cm) were placed at the bottom of the conical flask. Thereafter, the bacterial film formed on the glass chips present at the lower portion of the media was collected and then gently washed with sterilized PBS-1X twice, followed by dehydration using ethanol gradients (5–100% at 5% interval for 1 min each). Analyzed samples were subsequently mounted in the gold grid for 105 s and observed under SEM (GeminiSEM-450, Zeiss, UK) (150).

#### Siderophore production

Siderophore production of the gut isolate *A. veronii* CMF was determined on Chrome-azurol S (CAS) agar medium as per the method of Schwyn and Neilands (151). The bacterial strain CMF was grown on Luria-Bertani broth for 24 h, using 1% (vol/vol) overnight-grown culture of CMF as the inoculum. Further, CAS agar media were spotted (3 µL) with overnight-grown cultures and incubated at 37°C for 72 h.

#### Nitrogen fixation

To check the nitrogen-fixing capability of the gut isolate, CMF was grown on nitrogen-free medium (152). Here, overnight-grown CMF culture in LB broth was spotted (3 µL) in the N_2_-fixing medium (N-free medium) and incubated at 37°C for 24 h.

#### *In silico* plant growth-promoting potential of CMF

In search of PGP attributes of *A. veronii* CMF, proteins related to the phosphate solubilization, IAA production, and siderophore production were extracted from the whole-genome sequences of the strain CMF.

#### *In vivo* plant growth-promoting proficiencies of CMF

Plant growth-promoting potential of the CMF was accomplished with two model dicot and monocot plants *Cicer arietinum* L. and *Oryza sativa* L. IR36, respectively. To conduct this study, soil samples were obtained from the medicinal garden, Department of Botany, Visva-Bharati, India. On the other hand, the seed samples of the model plant were procured from the Department of Agronomy, Visva-Bharati, India. Phenotypic PGP pot experiments were featured with several parameters, viz., total length, root length, shoot length, stem diameter, estimation of chlorophyll and carotenoid, and plant root colonization potential of the gut isolate CMF in model plants. These were also documented upon gut bacterial potential in comparison to the control sets (without CMF).

##### Pot experiment to evaluate the plant growth-promoting phenotypes by CMF

Experimental seeds, e.g.*, C. arietinum* L. and *O. sativa* L. IR36, were surface sterilized with 3% sodium hypochlorite (NaOCl) solution, washed twice with sterile distilled water, and germinated in dark conditions for 72 h in sterilized Petri dishes lined with sterile moist blotting paper. Plastic pots (height × width = 8 × 4 cm) were prepared with sterilized soil for each plant samples in triplicate condition with respect to the CMF (*n* = 6). Germinated seedlings (3 days old) of equal size were planted in each pot after the treatment with fresh culture of CMF (OD at 600 nm = 0.5) by dipping the root systems for 30–40 min. A control set was also prepared with the sterilized LB broth instead of bacterial treatment. This experiment was accomplished in a completely randomized design at the departmental greenhouse garden during the winter season when the average temperature and sunlight period were around 20°C and 8–9 h, respectively, with sterile water applied as needed. After one week of plantation, bacterial cell suspensions as a dose of 3.8 × 10^5^ cells/gm soil in PBS-1X (pH 6.5) were applied to the soils of the tested pots.

##### Chlorophyll and carotenoid estimation

As a part of PGP attributes, the chlorophyll (Chl a, Chl b, and Chl a+ b) and total carotenoid (C_X+C_) contents of the gut bacteria treated and control sets of model plants in pot experiments were also evaluated according to the protocol of Porra (153) and Wellburn (154), respectively.

##### Plant root colonization potential of the gut isolate *A*. *veronii* CMF

*In vivo* plant root colonization potential of the gut isolate *A. veronii* CMF was carried out on the selected model plants, i.e., *C. arietinum* L. and *O. sativa* L. IR36, and this study was accomplished according to the protocol of Singh and Jha (95). To visualize the colonization of the gut isolates in tested plant root system, experimented root fragments (<0.3 cm) or root hairs were immersed in staining acridine orange (in 0.05% [wt/vol] 50 mM sodium acetate buffer [pH 5.0]) solution, followed by gentle wash three times with sterile double-distilled water. Then, the stained root portions were observed under a fluorescence microscope (FM) (Leica DM6 B Microsystems, Germany) at an intensity between 432 and 590 nm. Additionally, root samples were also prepared for SEM analysis according to Rovira and Campbell (155).

### Bioremediational attributes of CMF

#### *In vitro* bioremediational efficiency of CMF

Detailed *in vitro* heavy metal (HM) tolerant, removal, and accumulation efficiency (As^3+^, Cd^2+^, Cr^6+^, Cu^2+^, Pb^2+^, and Hg^2+^) of the gut isolate CMF was investigated in this study. Here, all the HMs used were sodium arsenite (NaAsO_2_), cadmium chloride (CdCl_2_), potassium dichromate (K_2_Cr_2_O_7_), copper sulfate (CuSO_4_), lead acetate [Pb(C_2_H_3_O_2_)_2_], and mercuric chloride (HgCl_2_) as a source of experimented HMs, respectively.

##### Heavy metal tolerant potential of CMF

The gut isolate CMF was tested against the above-mentioned HMs to characterize their minimum inhibitory concentrations (MICs) according to the protocol of Andrews (156). Overnight-grown bacterial culture (3.8 × 10^5^) in sterilized LB broth at 37°C with shaking condition (150 rpm) was spread on the NA plates. Then, the sterilized paper discs (5 mm in diameter of Whatman No. 1 filter paper) were soaked in the specific concentration (300, 500, 1,000, 1200, 1250, 1300, and 1500 µg/mL) of each experimented HMs and placed on the agar plates. Thereafter, the plates were incubated at 37°C for 24 h and subjected to documentation. A control set was also prepared for each experiment where discs were soaked with the sterile deionized water.

##### Heavy metal removal efficiency of CMF

In accordance with the HM tolerance characteristic of the gut isolate CMF, its highest HM removal proficiency was assessed through atomic absorption spectroscopic (AAS) analysis. In this study, the strains were grown in sterilized LB broth (prepared in deionized water) with different HM concentrations (10, 100, and 1,000 µg/mL) at 37°C for 72 h under shaking conditions (100 rpm). After the incubation, liquid cultures were centrifuged at 10,000 rpm for 10 min, and the CFS was collected. The CFS of each sample was then filtered through a bacterial filter to eliminate residual cell debris, and the filtrates were analyzed in AAS (Perkin-Elmer PinAAcle 900F, USA). A control set of the experiment was also prepared for the AAS analysis without any HMs in sterile LB broth along with the same other factors. HM removal potential of the gut isolate CMF was calculated according to the modified formula of Pandey and Bhatt (157). Here, IC represents the initial HM concentration (µg/mL) given in the culture medium at time zero, and FC represents the final concentration (µg/mL) of HM after the incubation period.


(2) $$HM\;Removal=\frac{IC-FC}{IC}\times 100$$

##### Heavy metal accumulation potential of CMF

To evaluate the HM accumulation ability of the gut symbiont CMF, SEM-derived energy-dispersive X-ray spectroscopic (EDXS) analysis (GeminiSEM-450, Zeiss, UK) was performed according to the protocol of Hartmann et al. (158) with minor modifications. In this study, bacterial strains were grown in LB broth (prepared in deionized water) with HMs like As^3+^, Cu^2+^, and Cr^6+^ (1 mg/mL) at 37°C for 24 h under shaking conditions (100 rpm). A control set was also prepared without the addition of any HMs in the culture medium. After the incubation, liquid cultures were centrifuged at 10,000 rpm for 10 min, and the CFS was discarded, followed by the cell pellets collection. Harvested cells were washed thrice with PBS-1X (pH 7.0) and fixed with 2.5% glutaraldehyde (wt/vol) in PBS-1X (pH 7.0) for overnight at 4°C. After that, the cell pellets were again washed with PBS-1X (pH 7.0) and passed through the ethanol gradation (30–100%) with 1-minute interval for each gradation. Analyzed samples were subsequently mounted in the gold grid for 105 s (Quorum, SC76220, Sputter coater) and observed under SEM-EDXS (GeminiSEM-450, Zeiss, UK) to detect elemental composition within the experimented cells.

### *In silico* bioremediational attributes of CMF

In search of bioremediational attributes of *A. veronii* CMF, detoxifying genes, proteins, chaperones, and transport systems related to the heavy metal resistance/tolerance and accumulation were extracted from the whole-genome sequences.

## Data Availability

Data will be made available on request.

## SUPPLEMENTAL MATERIAL

Graphical abstract spectrum.01622-25-s0001.tif Visual depiction of the study.

Supplemental material spectrum.01622-25-s0002.pdf Figures S1 to S19; Tables S1 to S8.

ASM does not own the copyrights to Supplemental Material that may be linked to, or accessed through, an article. The authors have granted ASM a non-exclusive, world-wide license to publish the Supplemental Material files. Please contact the corresponding author directly for reuse.
