# Supplementary figures and images for: Biocontrol, plant growth-promoting, and bioremediation potential of Aeromonas veronii CMF from the gut of Chrysomya megacephala
Source: Microbiol Spectr. 2025 Nov 4;13(12):e01622-25. doi: 10.1128/spectrum.01622-25 (PMC12671090; doi:10.1128/spectrum.01622-25)

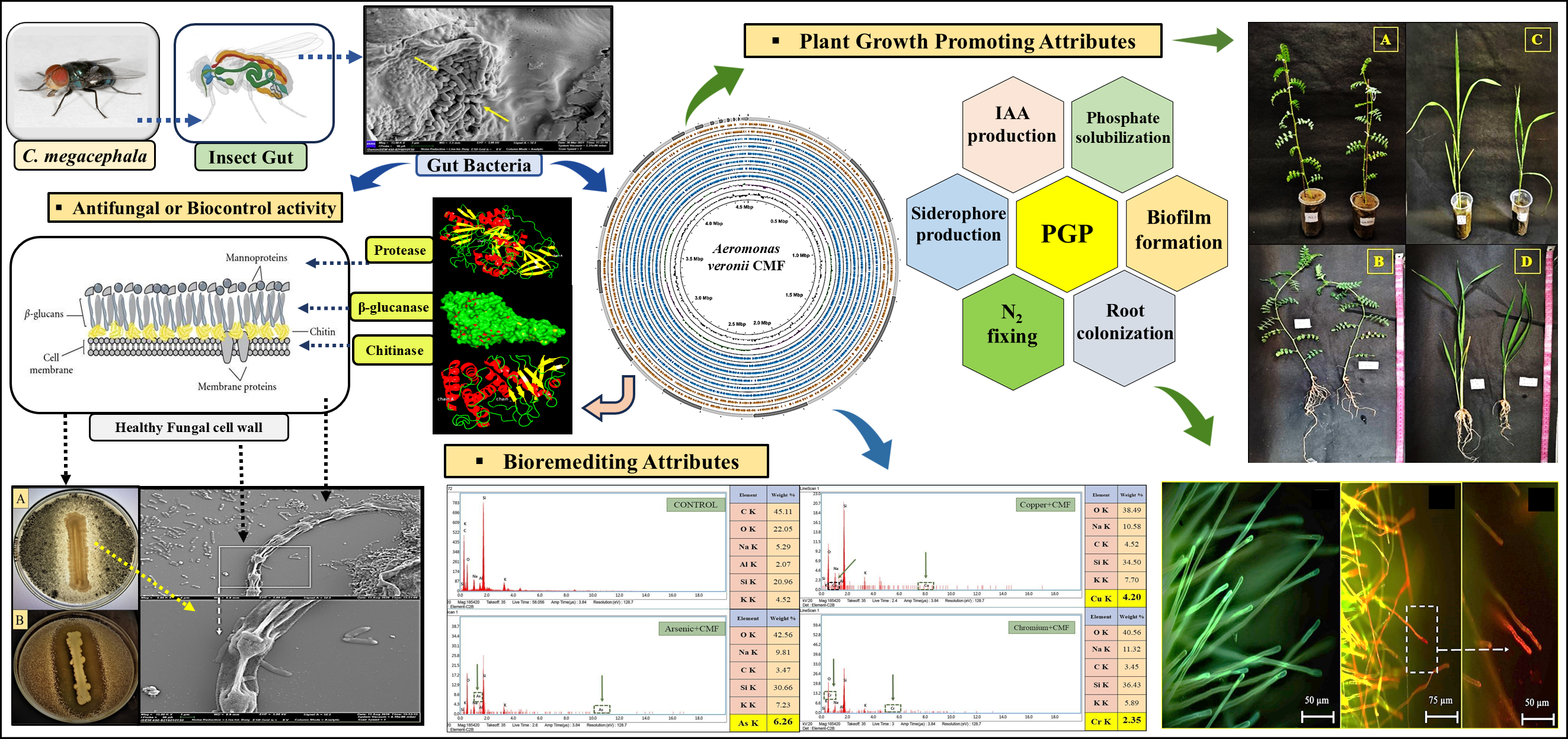

Supplement: Graphical abstract — Visual depiction of the study. [file spectrum.01622-25-s0001.tif]
