# Supplementary material for: Biocontrol, plant growth-promoting, and bioremediation potential of Aeromonas veronii CMF from the gut of Chrysomya megacephala
Source: Microbiol Spectr. 2025 Nov 4;13(12):e01622-25. doi: 10.1128/spectrum.01622-25 (PMC12671090; doi:10.1128/spectrum.01622-25)
Supplement: Supplemental material — Figures S1 to S19; Tables S1 to S8. [file spectrum.01622-25-s0002.pdf]

Supplementary file to

Biocontrol, PGP, and Bioremediation Potential of *Aeromonas veronii* CMF from the gut of *Chrysomya megacephala*

Sandipan Banerjee[1,2,7*], Kunal Kumar Saha[1#], Krishnendu Pramanik[3#], Raju Biswas[4,5], Moumita Parveen[1], Srinivasan Balachandran[6], Hynek Roubík[7], Narayan Chandra Mandal[1*]

[1]*Mycology and plant pathology laboratory, Department of Botany, Visva Bharati University, Santiniketan-731235, West Bengal, India*

[2]*Faculty of Forestry and Wood Sciences, Czech University of Life Sciences Prague, Kamýcká 129, Prague 165 00, Czech Republic*

[3]*Department of Botany, Cooch Behar Panchanan Barma University, Panchanan Nagar, Vivekananda Street, Cooch Behar - 736101, West Bengal, India*

[4]*Microbiology Laboratory, Department of Botany, Visva Bharati University, Santiniketan-731235, West Bengal, India*

5Ecosystems Laboratory, Centre for Ecological Sciences (CES), Indian Institute of Science, Bengaluru, Karnataka, 560012, India

[6]*Bioenergy Laboratory, Department of Environmental Studies, Institute of Science, Visva-Bharati, Santiniketan-731235, India*

[7]*Department of Sustainable Technologies, Faculty of Tropical AgriSciences, Czech University of Life Sciences Prague, Kamýcká 129, 165 00 Prague, Czech Republic*

[#]Equally contributed as second author

Corresponding authors:

Prof. Narayan Chandra Mandal
E-mail: mandalnc@visva-bharati.ac.in
ORCID ID: 0000-0003-1631-0794

Dr. Sandipan Banerjee
Email: banerjee@fld.czu.cz
ORCID ID: 0000-0002-2102-8809

Supplementary figure

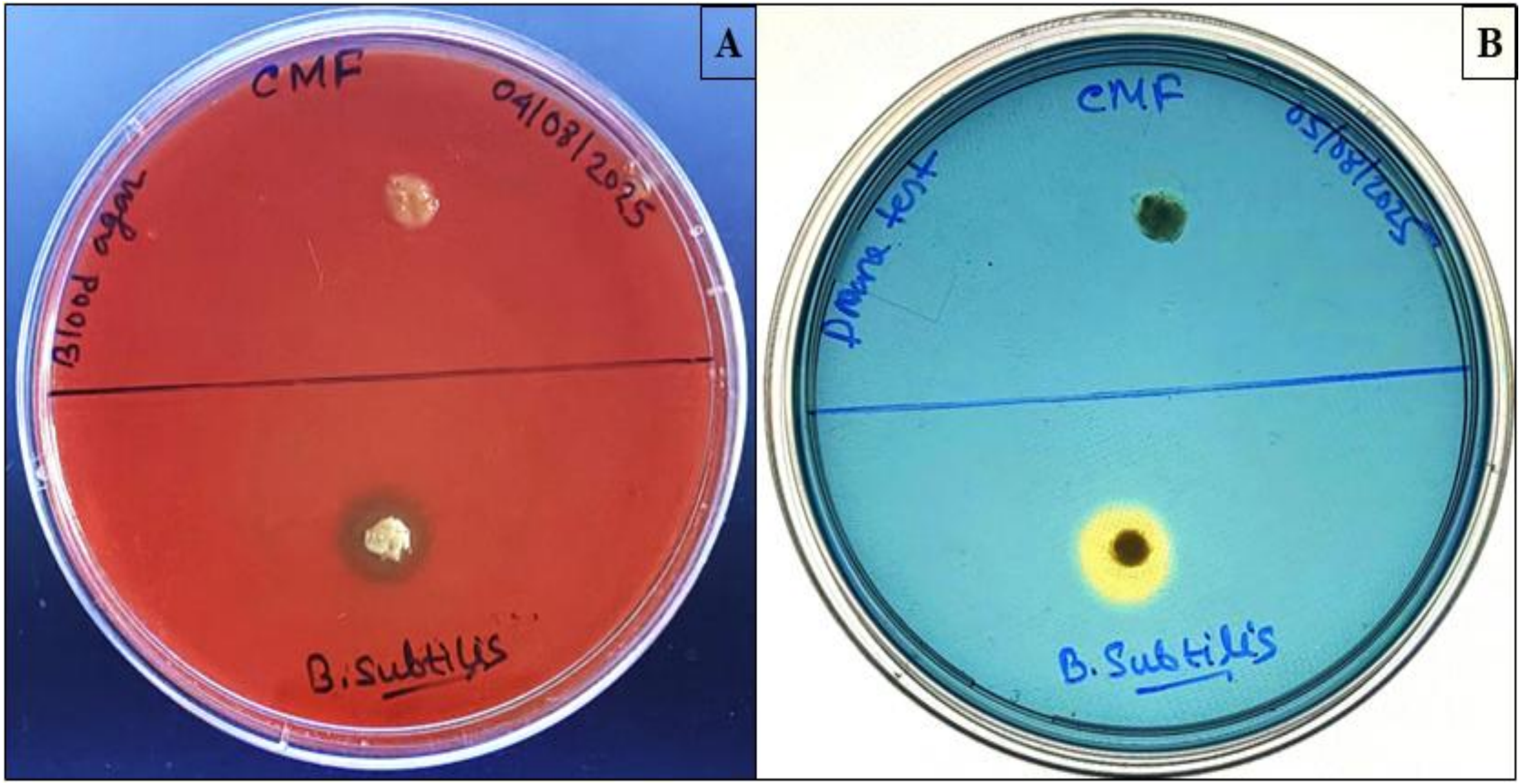

Supplementary figure. S1. Pathogenicity test of *A. veronii* CMF and *B. subtilis* MTCC 121 in (A) blood agar and (B) DNase agar plate

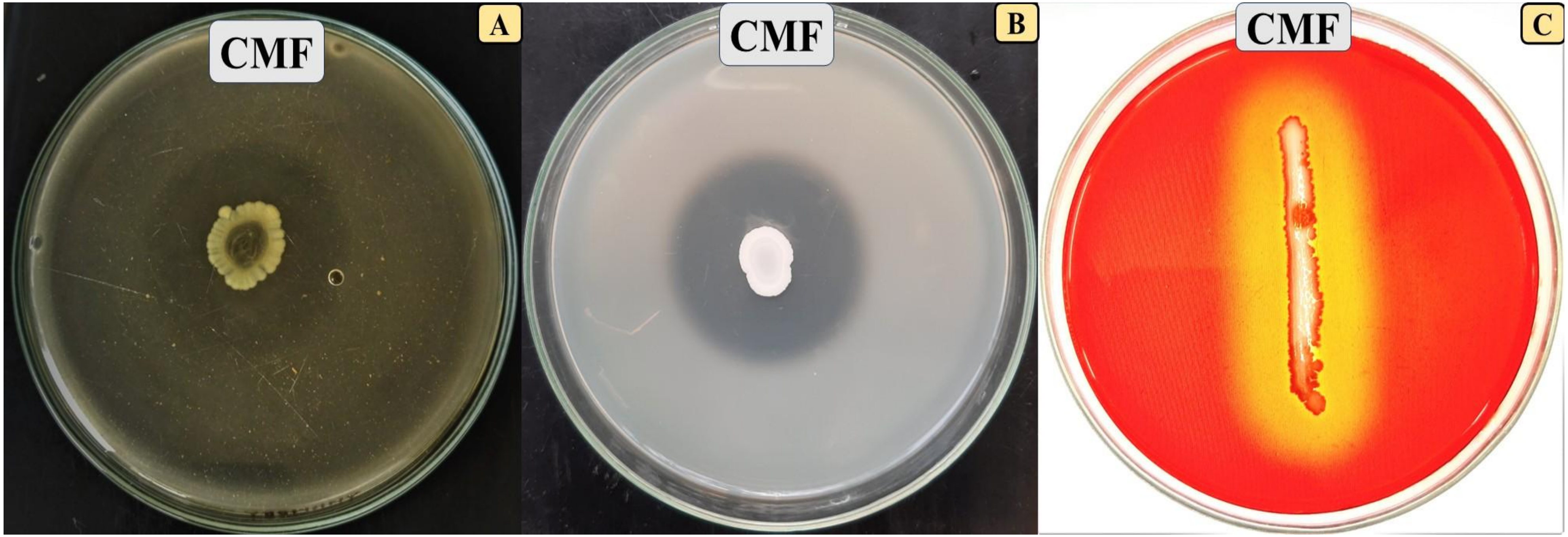

Supplementary figure. S2. Antifungal enzyme producing capability of *A. veronii* CMF (A) Chitinase (B) Protease (C) β-glucanase

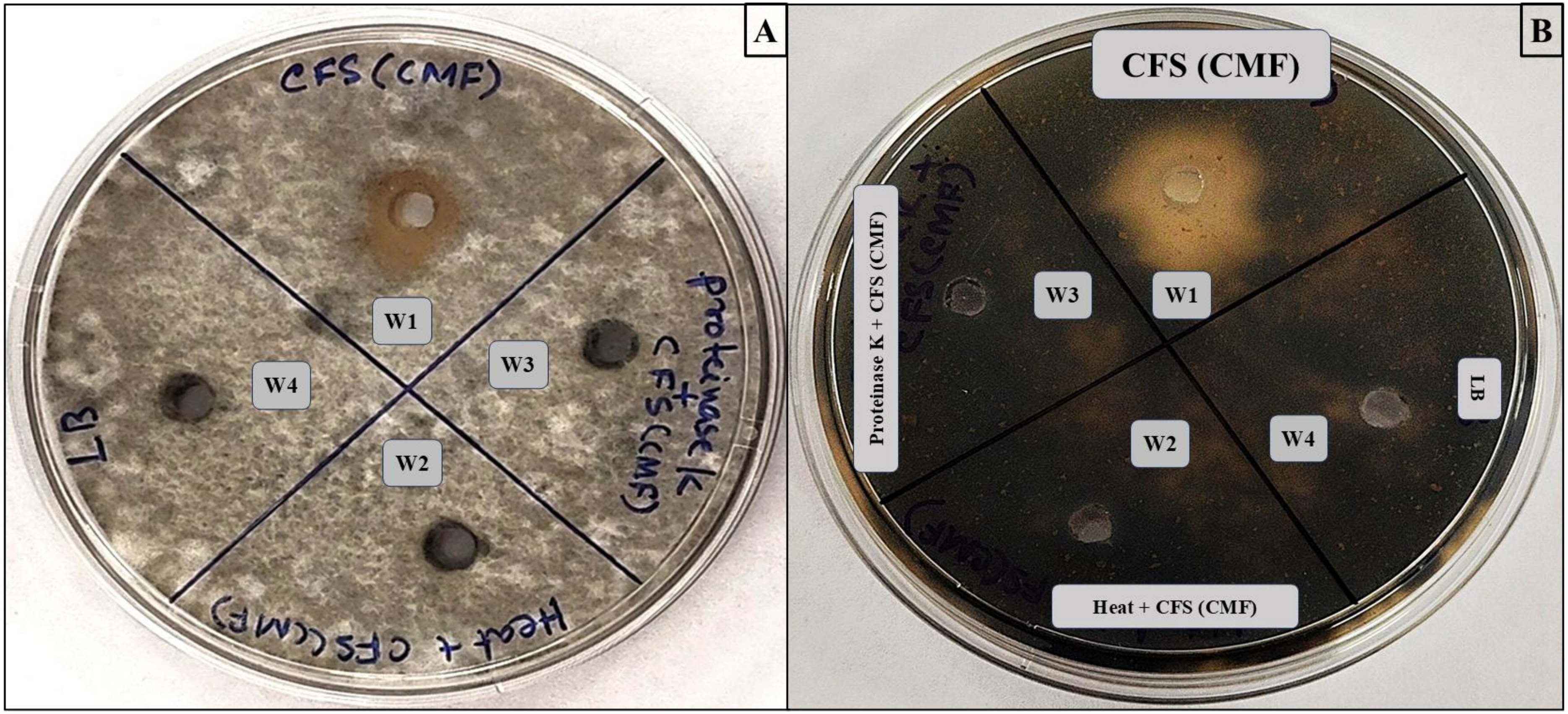

Supplementary figure. S3. Determination of the nature of antifungal metabolites produced by CMF against *Alternaria alternata* VBAV007. (A) Upside view of the experimented petri plate (B) Downside view of the experimented petri plate.

Well numbers signify the following: W1= CFS of CMF; W2= boiled CFS or heat killed CFS; W3= CFS treated with Proteinase K; W4= control set or sterilized medium (Luria Bertani broth)

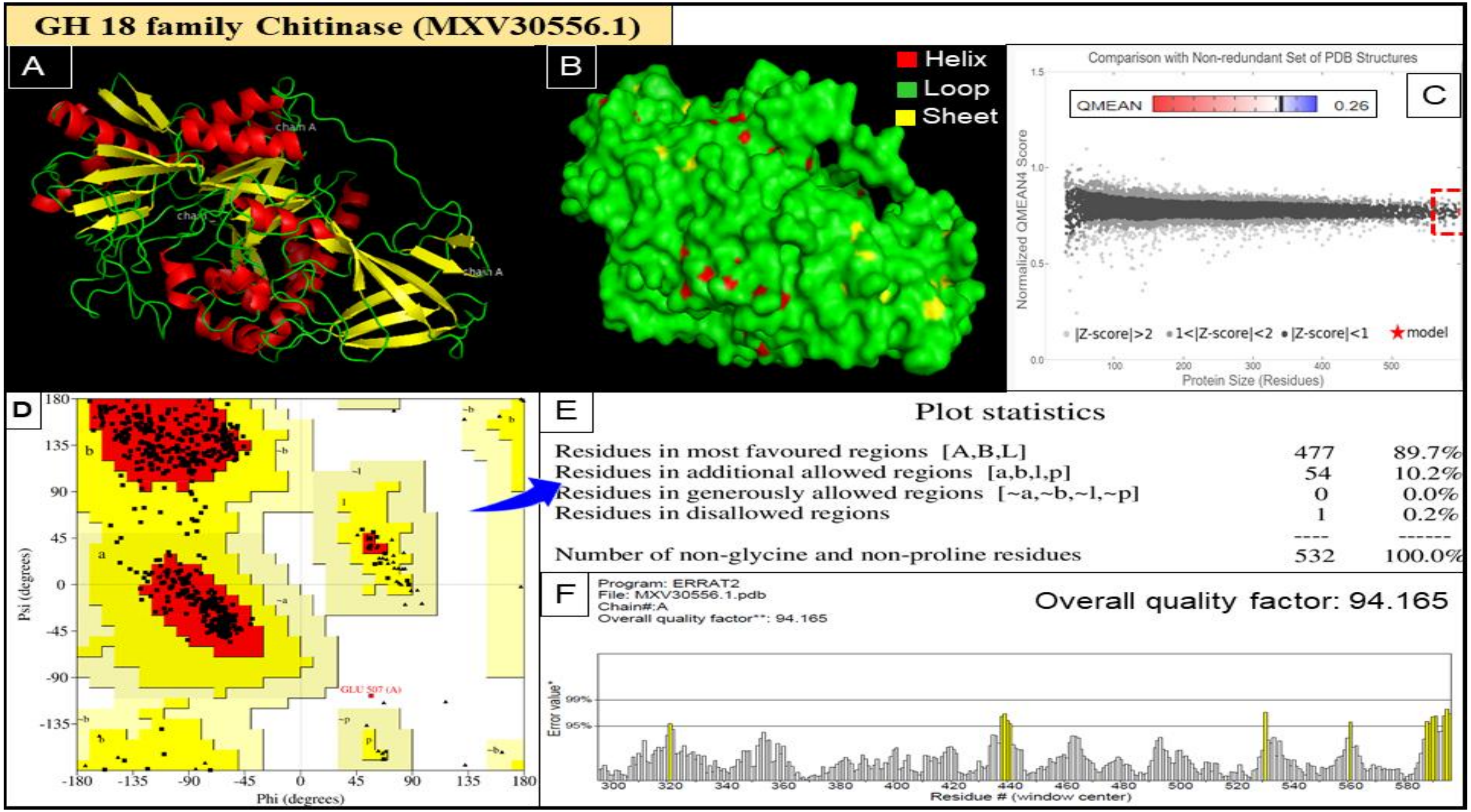

Supplementary figure. S4. Homology modeling (A-B), structural assessment (C-E), validation (F) of the GH 18 chitinase (MXV30556.1) identified from *A. veronii* CMF genome

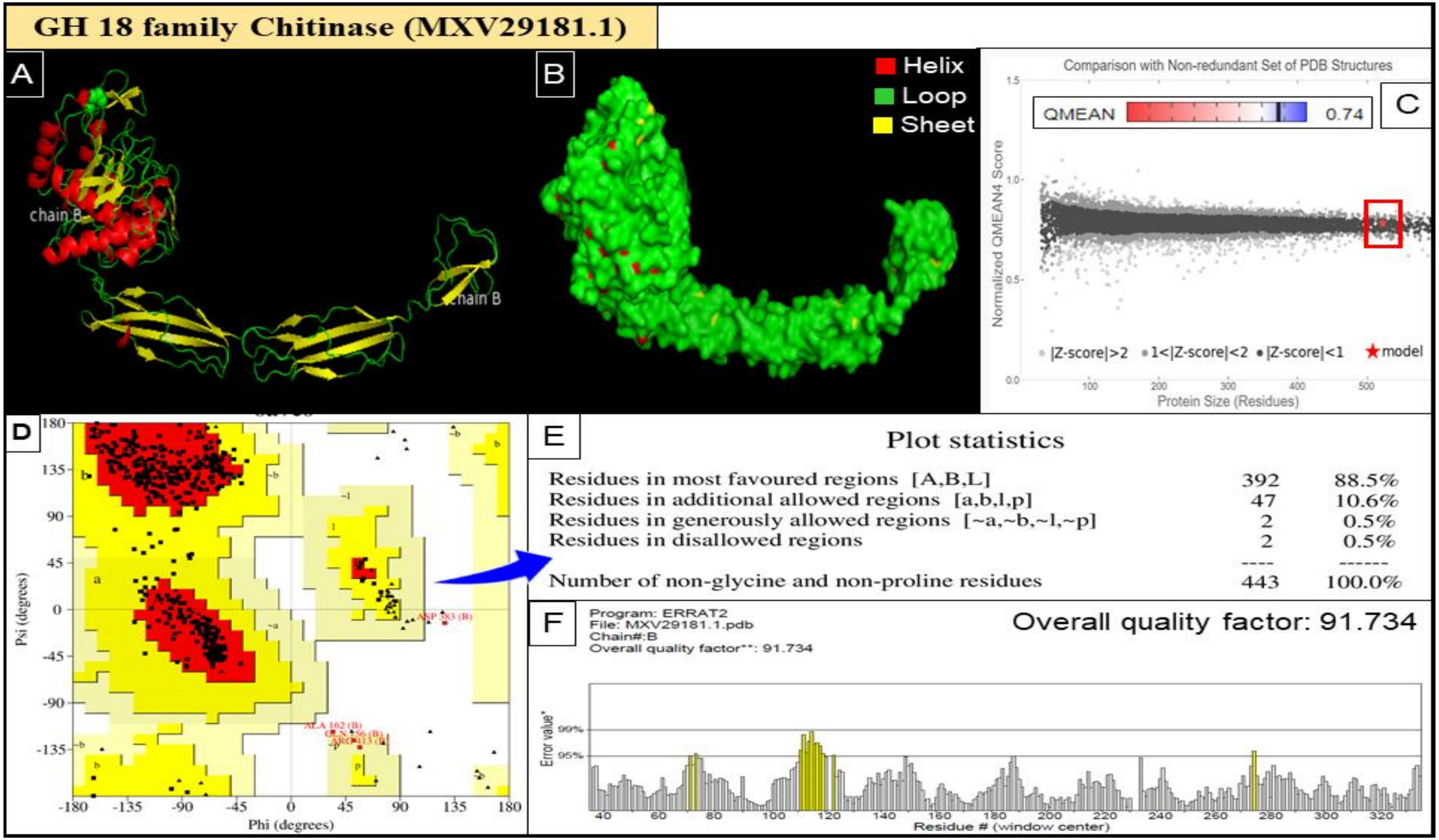

Supplementary figure. S5. Homology modeling (A-B), structural assessment (C-E), validation (F) of the GH 18 chitinase (MXV29181.1) identified from *A. veronii* CMF genome

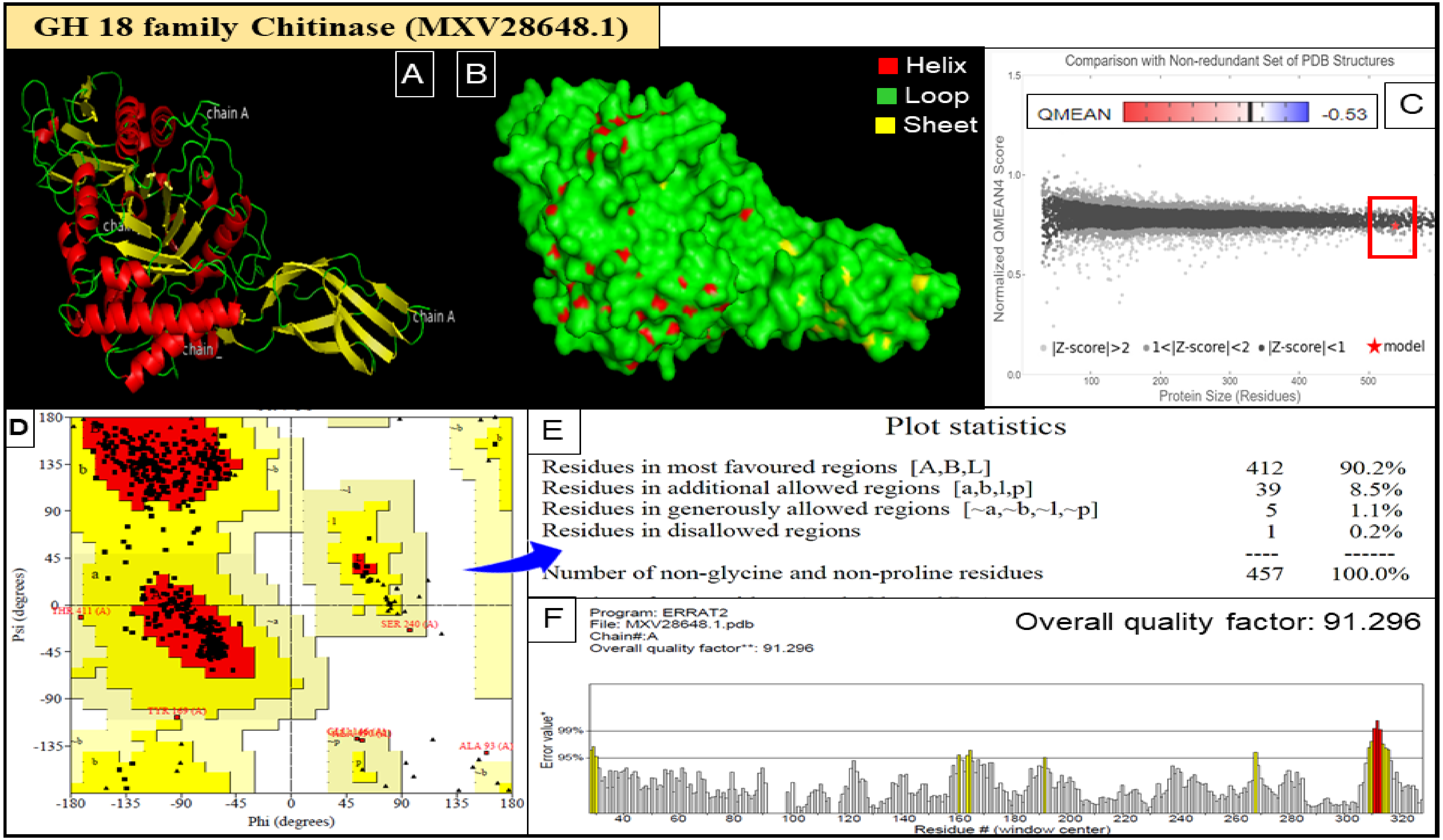

Supplementary figure. S6. Homology modeling (A-B), structural assessment (C-E), validation (F) of the GH 18 chitinase (MXV28648.1) identified from *A. veronii* CMF genome

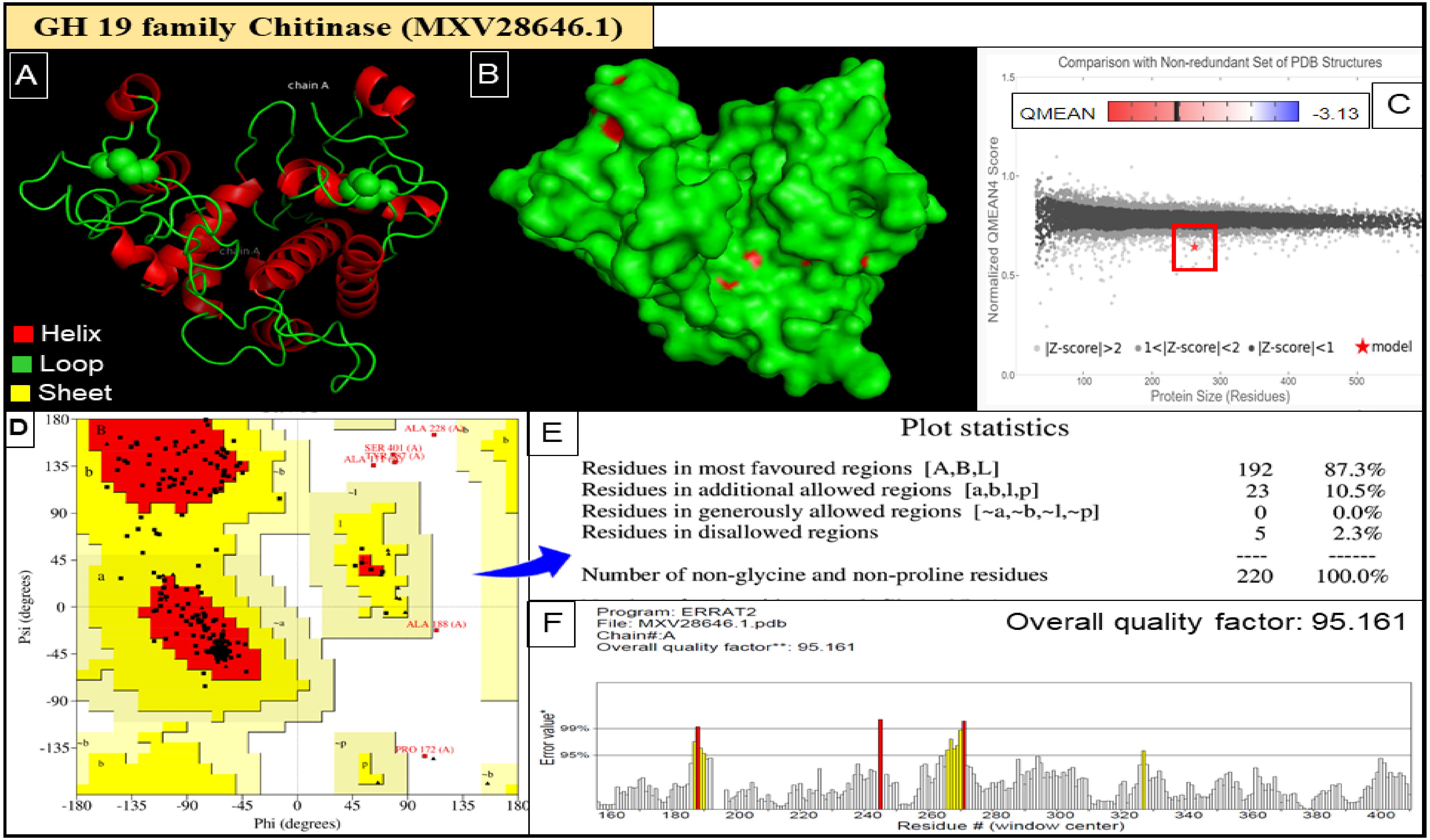

Supplementary figure. S7. Homology modeling (A-B), structural assessment (C-E), validation (F) of the GH 19 chitinase (MXV28646.1) identified from *A. veronii* CMF genome

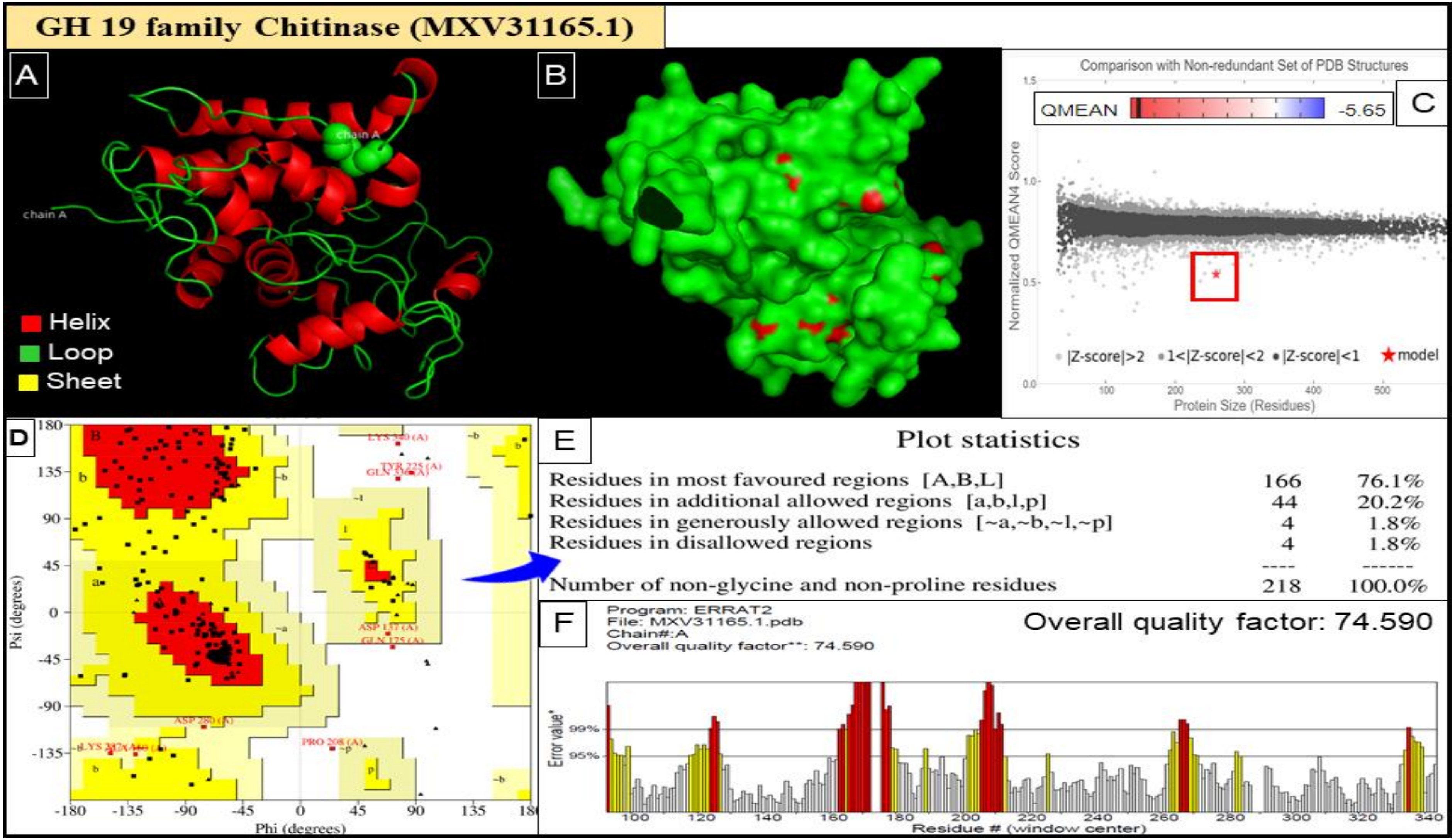

Supplementary figure. S8. Homology modeling (A-B), structural assessment (C-E), validation (F) of the GH 19 chitinase (MXV31165.1) identified from *A. veronii* CMF genome

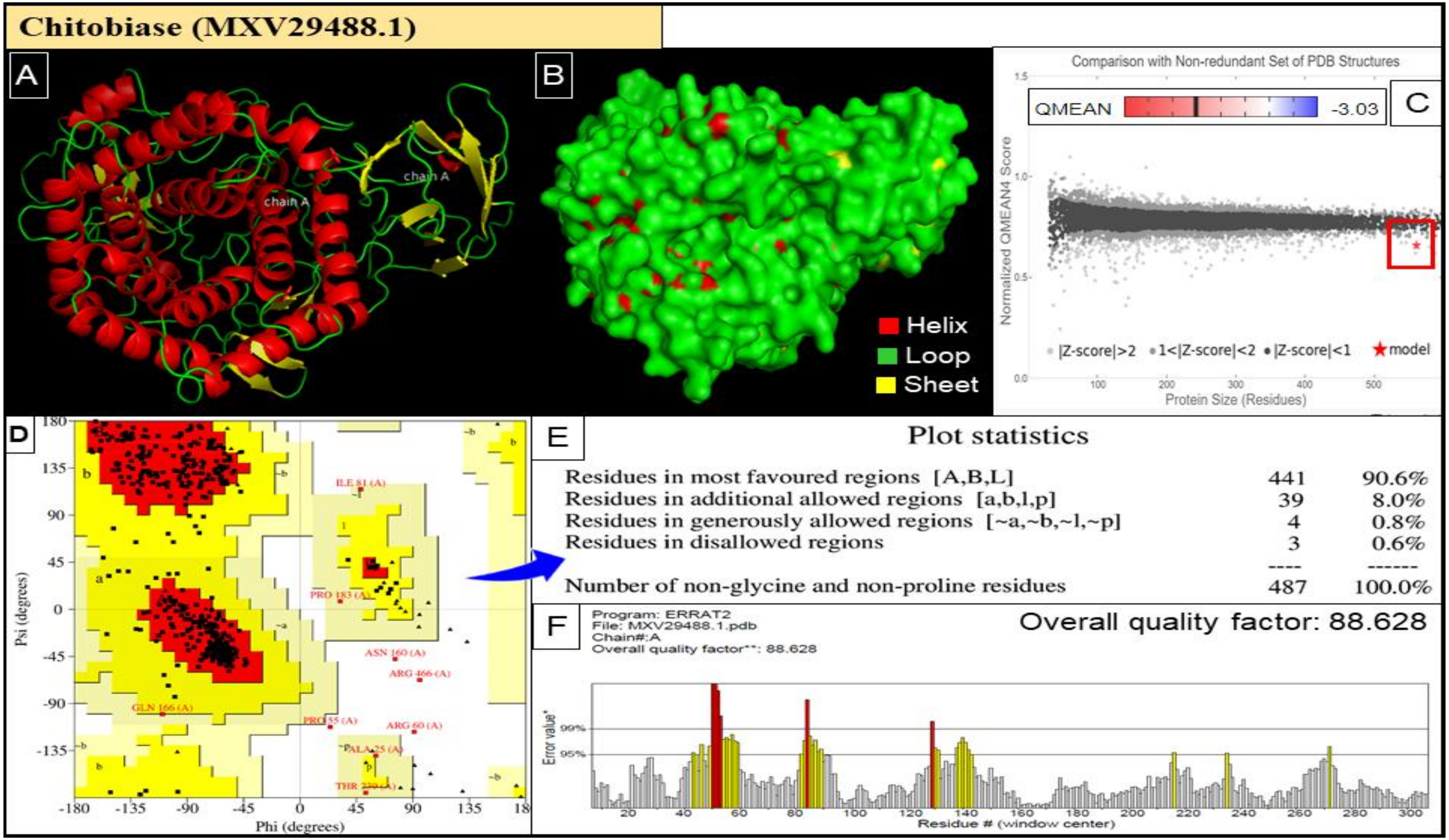

Supplementary figure. S9. Homology modeling (A-B), structural assessment (C-E), validation (F) of the Chitobiase (MXV29488.1) identified from *A. veronii* CMF genome

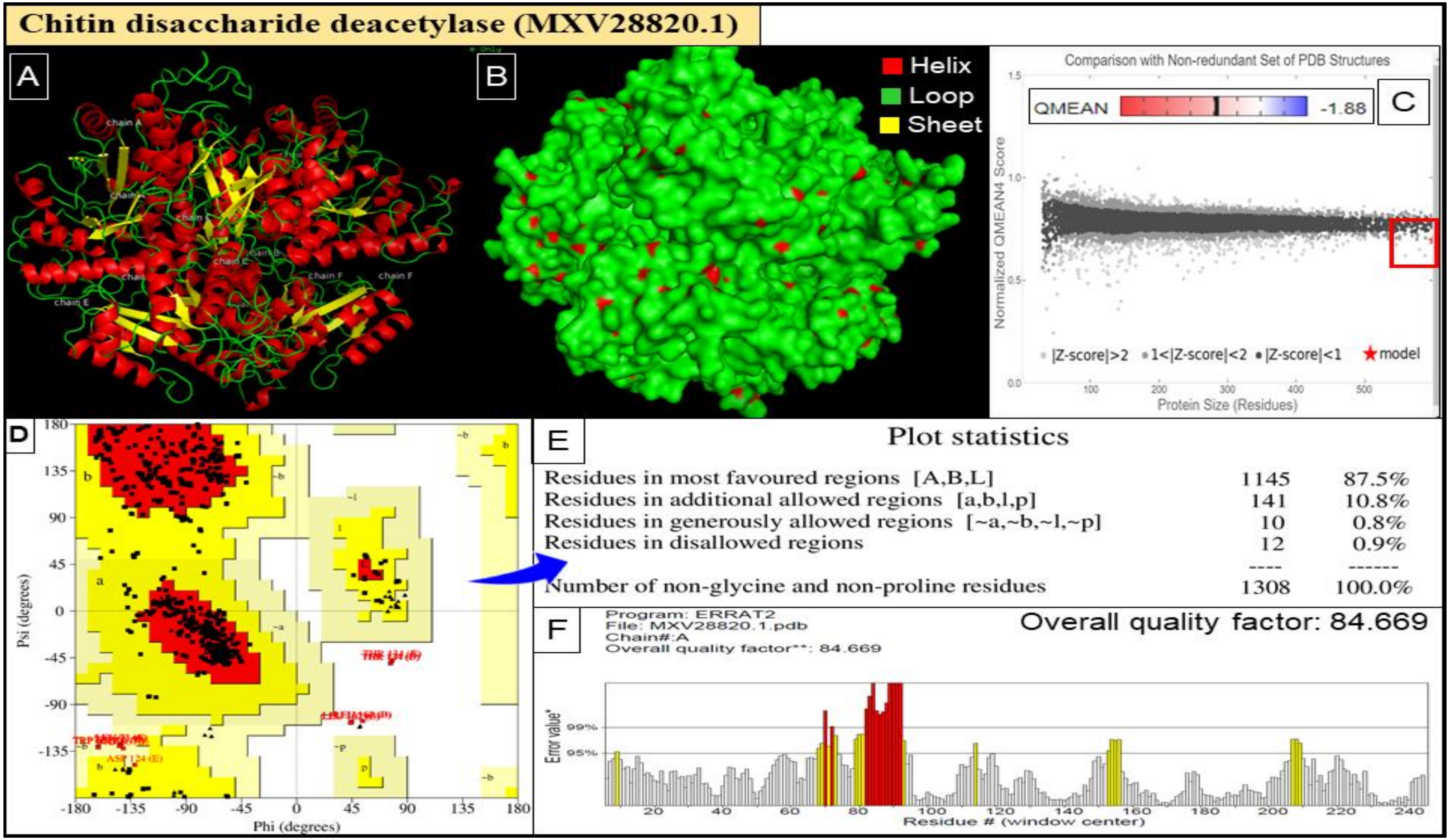

Supplementary figure. S10. Homology modeling (A-B), structural assessment (C-E), validation (F) of the Chitin disaccharide deacetylase (MXV28820.1) identified from *A. veronii* CMF genome

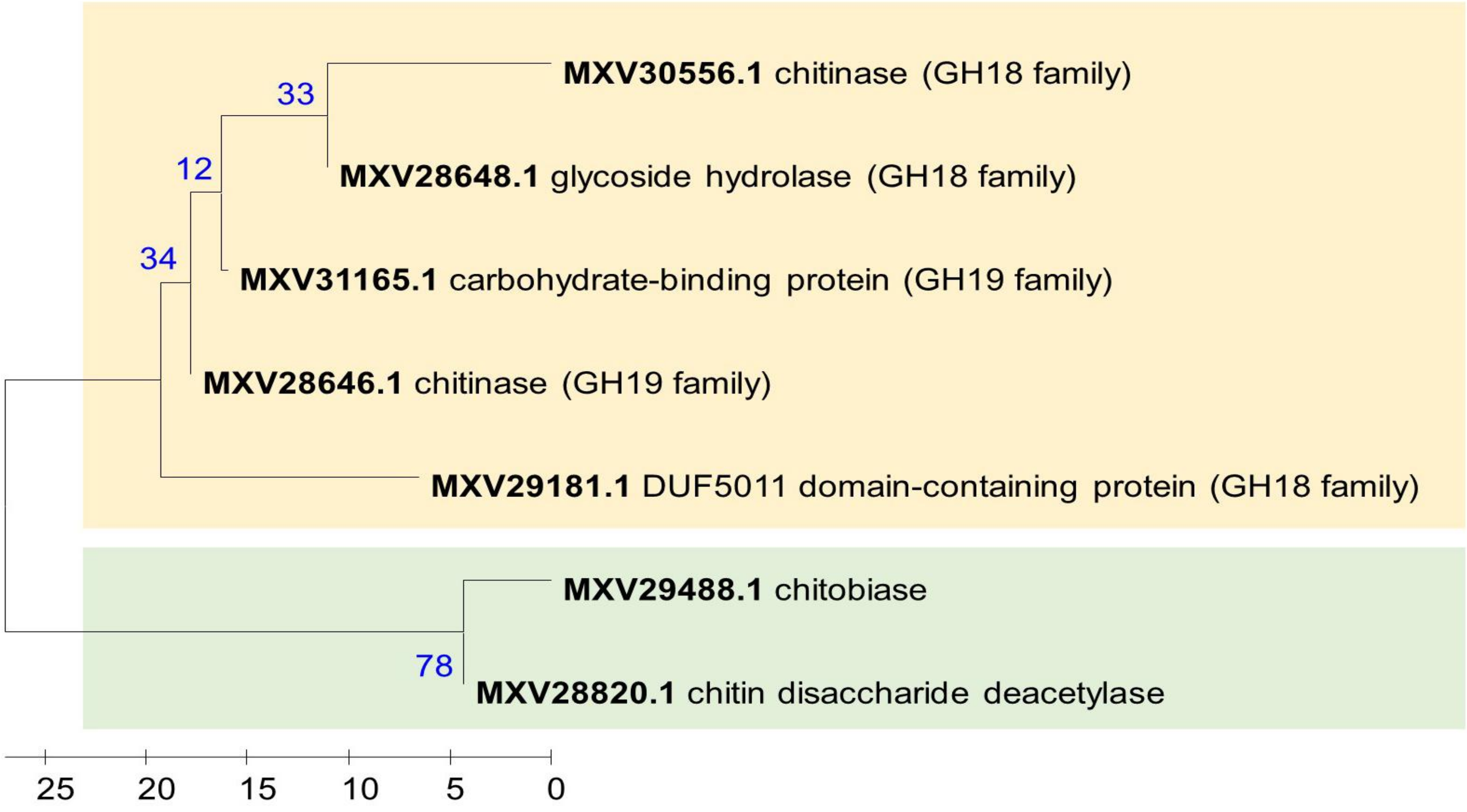

Supplementary figure. S11. Evolutionary relationship between GH 18 and GH 19 family chitinases from *A. veronii* CMF genome

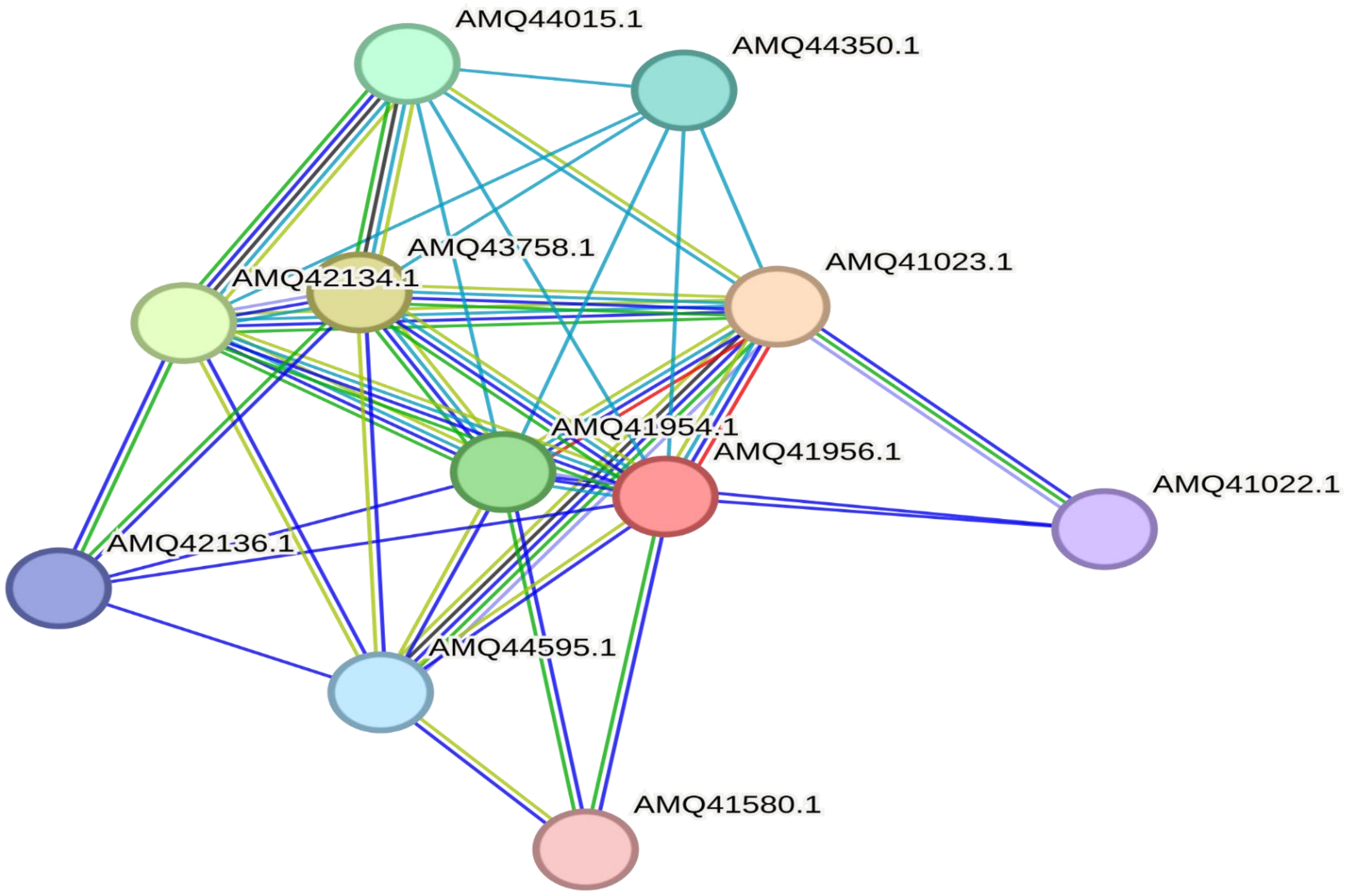

Supplementary figure. S12. Protein-protein interaction network showing predicted functional partners related to GH18: MXV28648.1 glycoside hydrolase [*Aeromonas veronii* CMF]

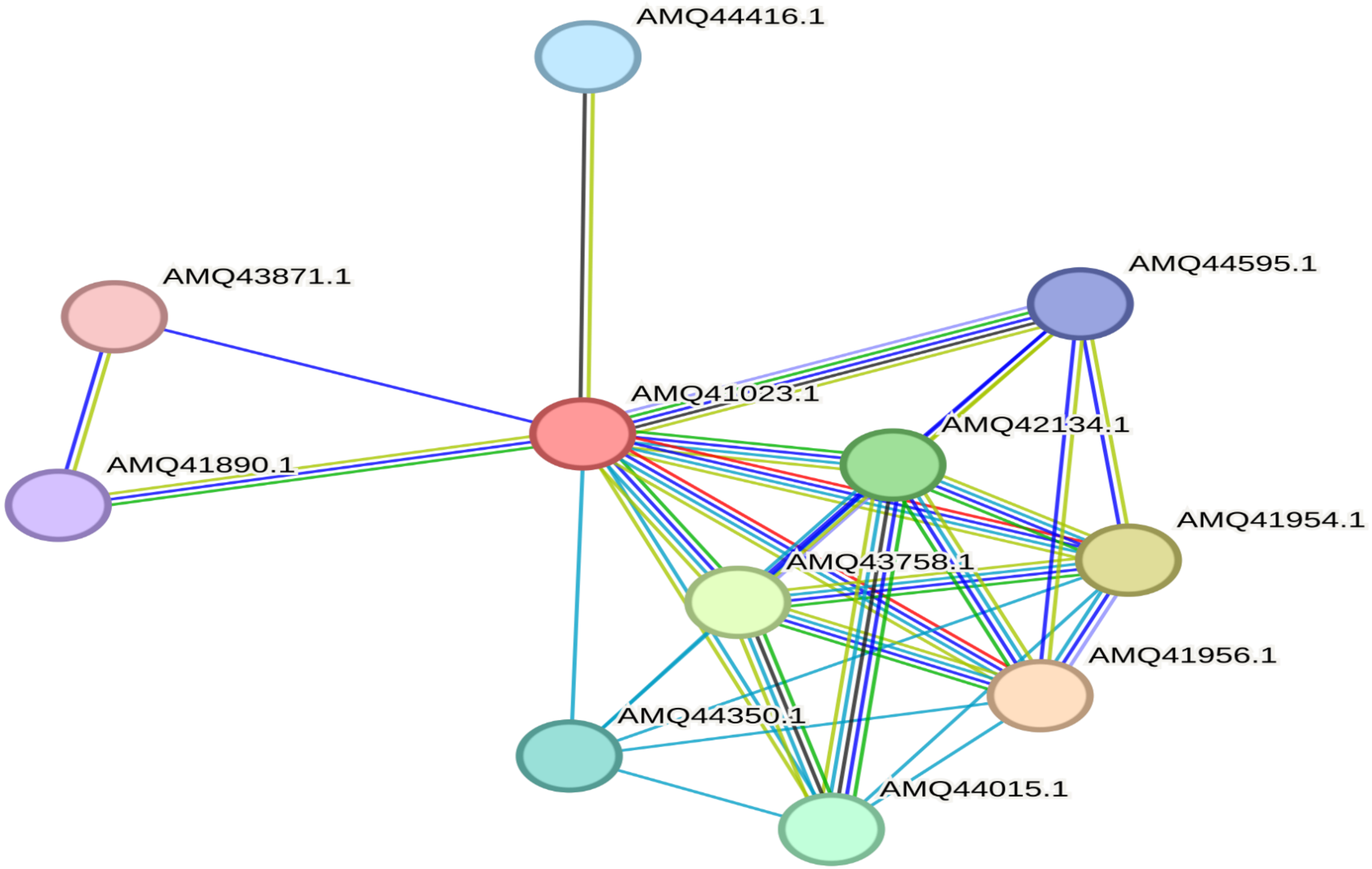

Supplementary figure. S13. Protein-protein interaction network showing predicted functional partners related to GH18: MXV29181.1 DUF5011 domain-containing protein [*Aeromonas veronii* CMF]

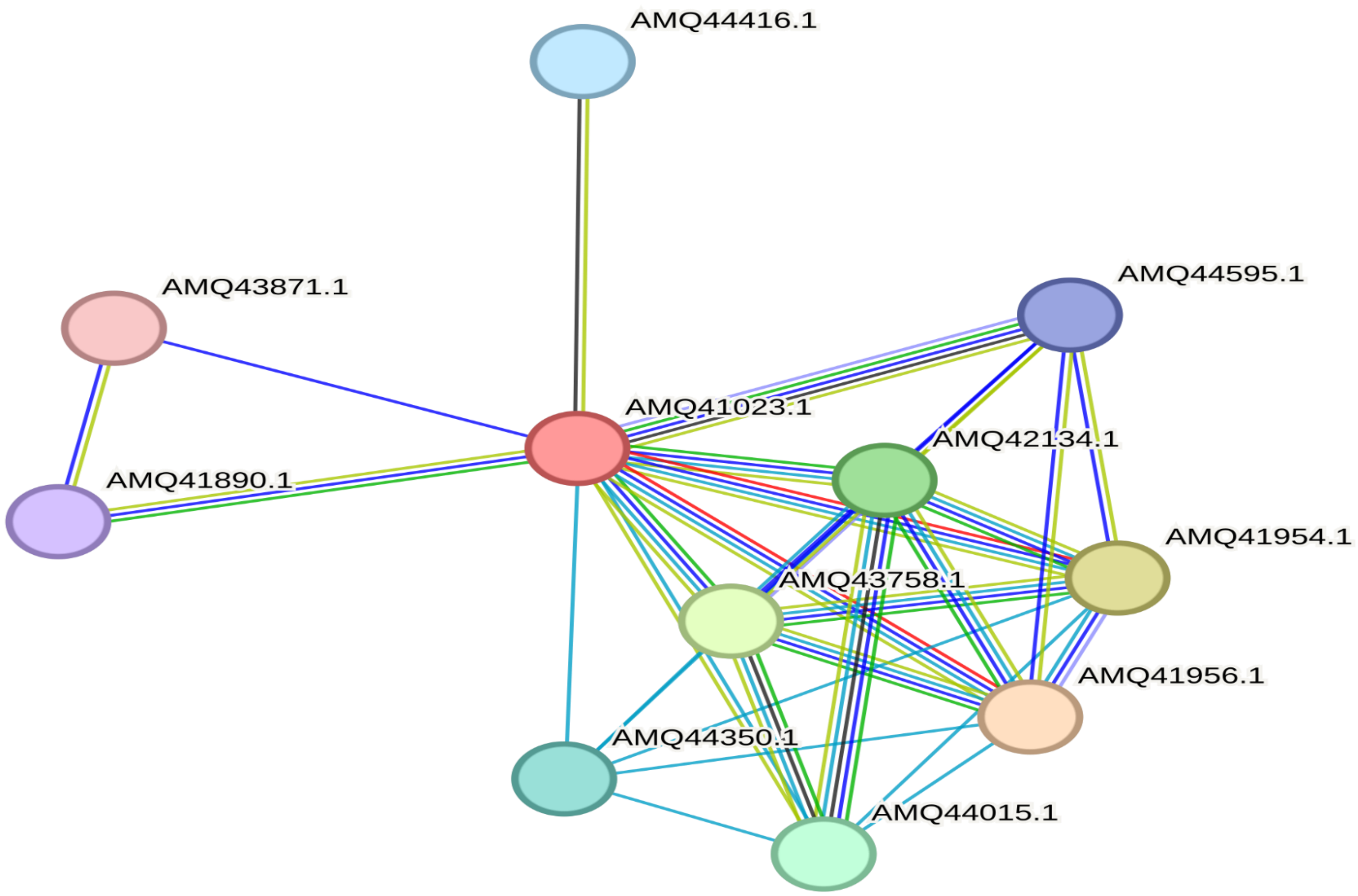

Supplementary figure. S14. Protein-protein interaction network showing predicted functional partners related to GH18: MXV30556.1 chitinase [*Aeromonas veronii* CMF]

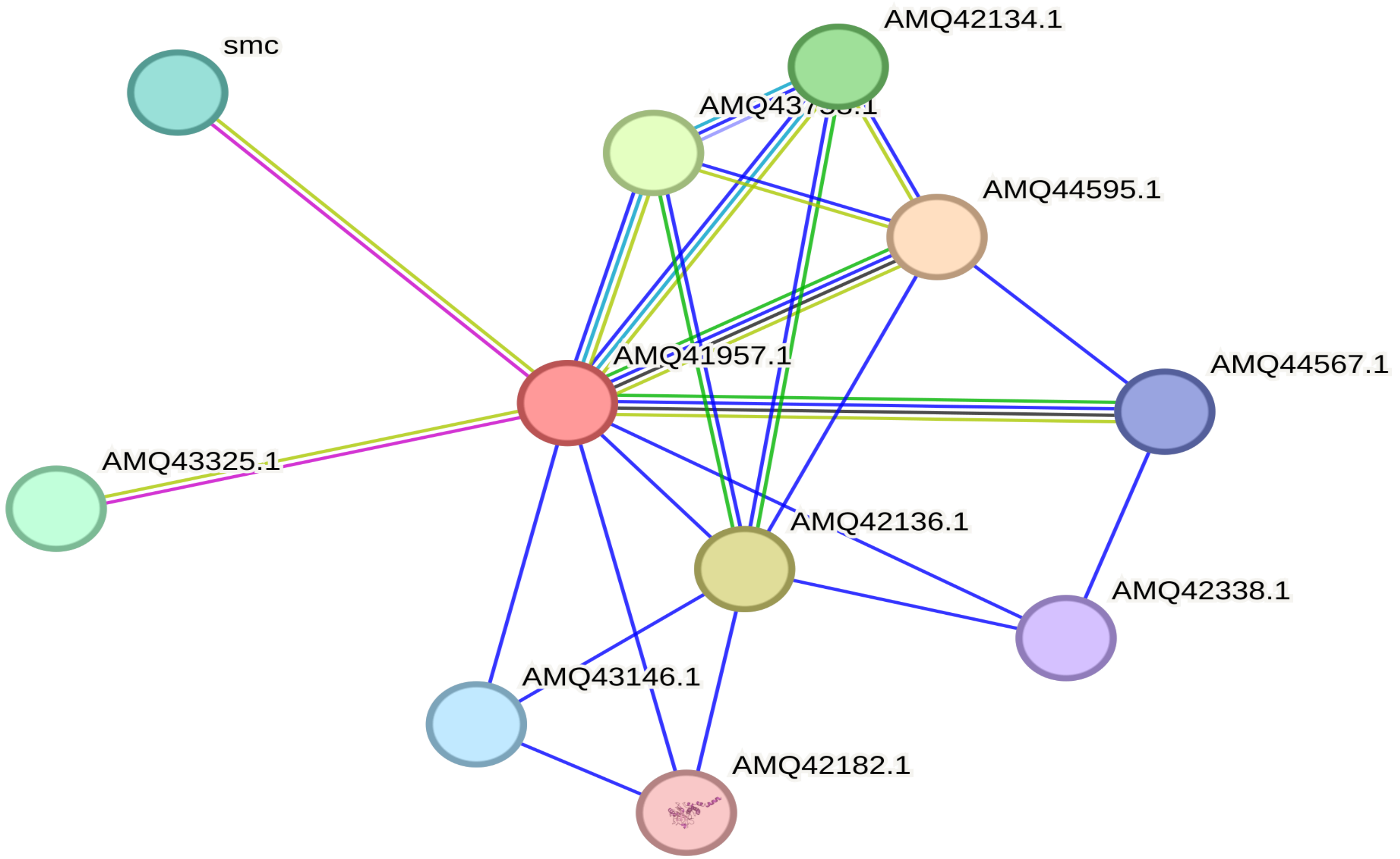

Supplementary figure. S15. Protein-protein interaction network showing predicted functional partners related to GH19: MXV28646.1 chitinase [*Aeromonas veronii* CMF]

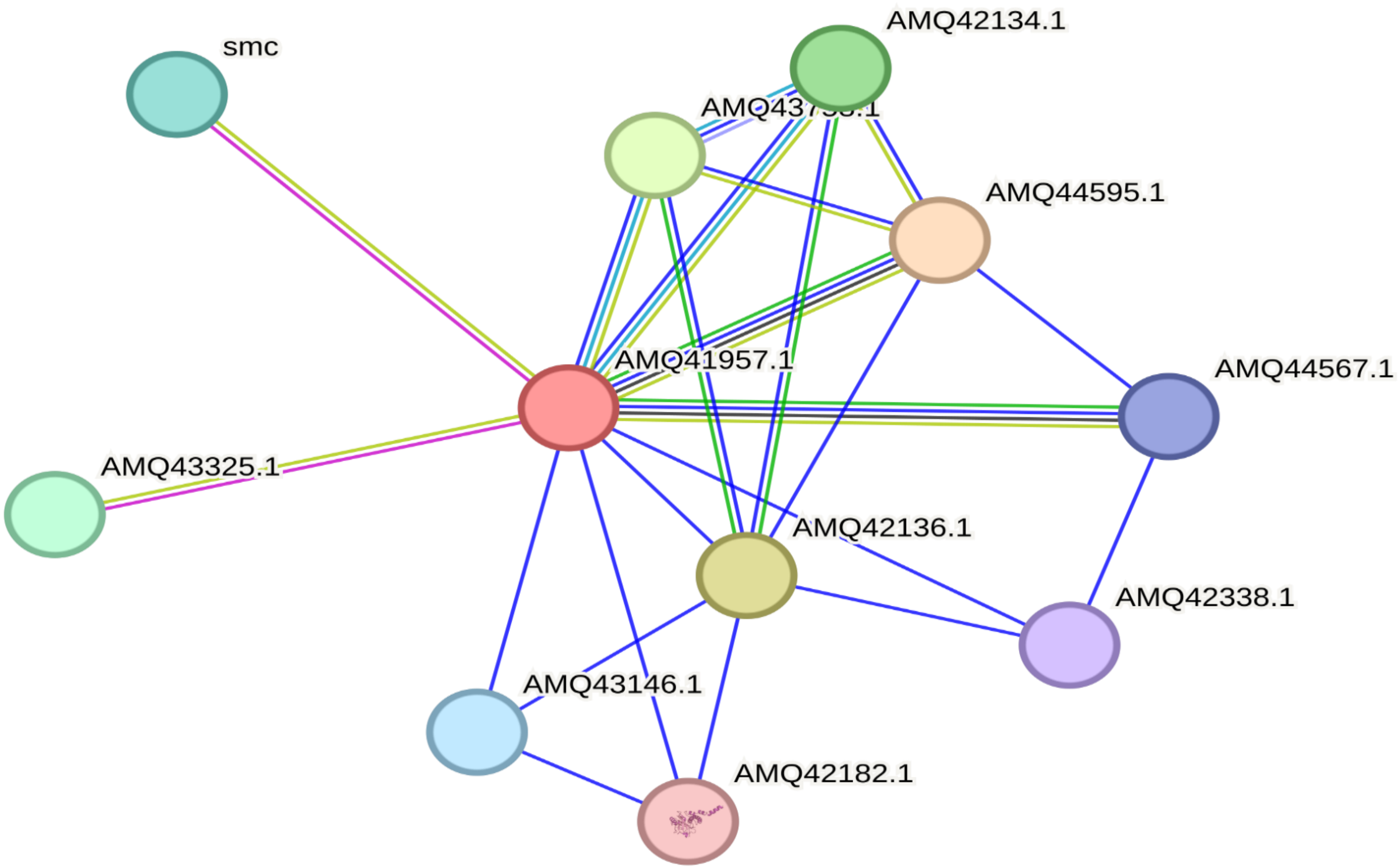

Supplementary figure. S16. Protein-protein interaction network showing predicted functional partners related to GH19: MXV31165.1 carbohydrate-binding protein [*Aeromonas veronii* CMF]

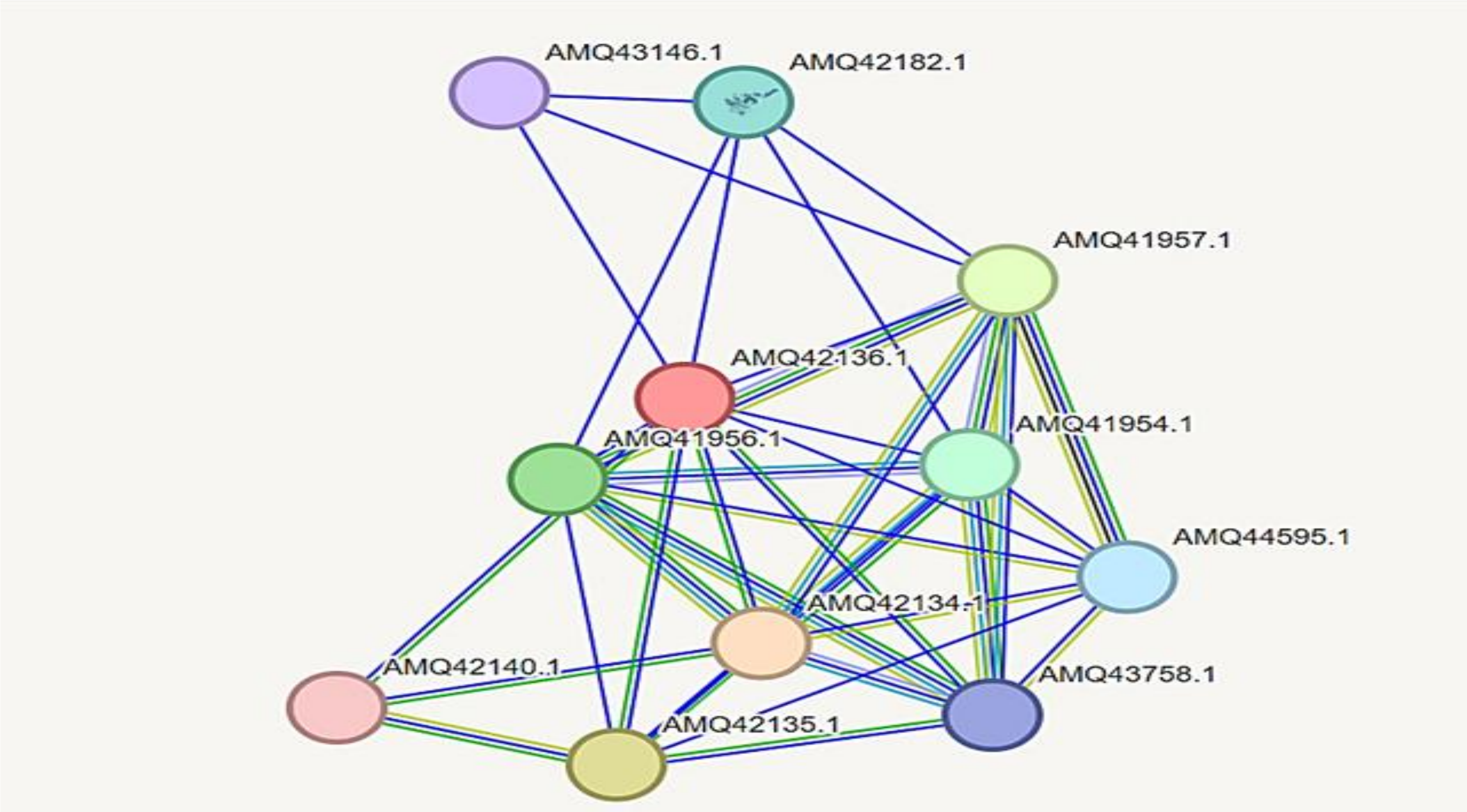

Supplementary figure. S17. Protein-protein interaction network showing predicted functional partners related to MXV29488.1 chitobiase [*Aeromonas veronii* CMF]

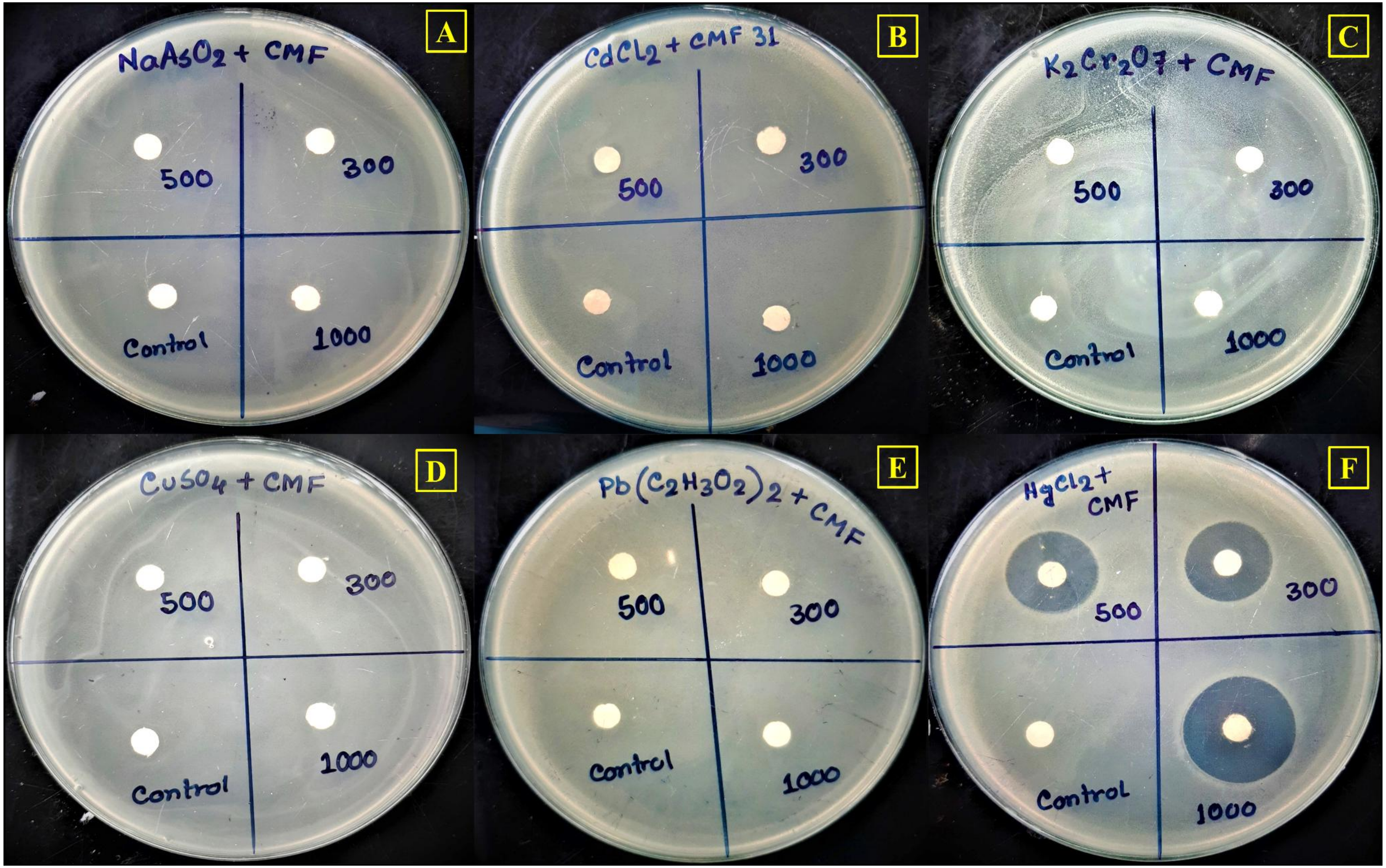

Supplementary figure. S18. Heavy metal resistant/tolerant potentiality of the gut isolate *Aeromonas veronii* CMF

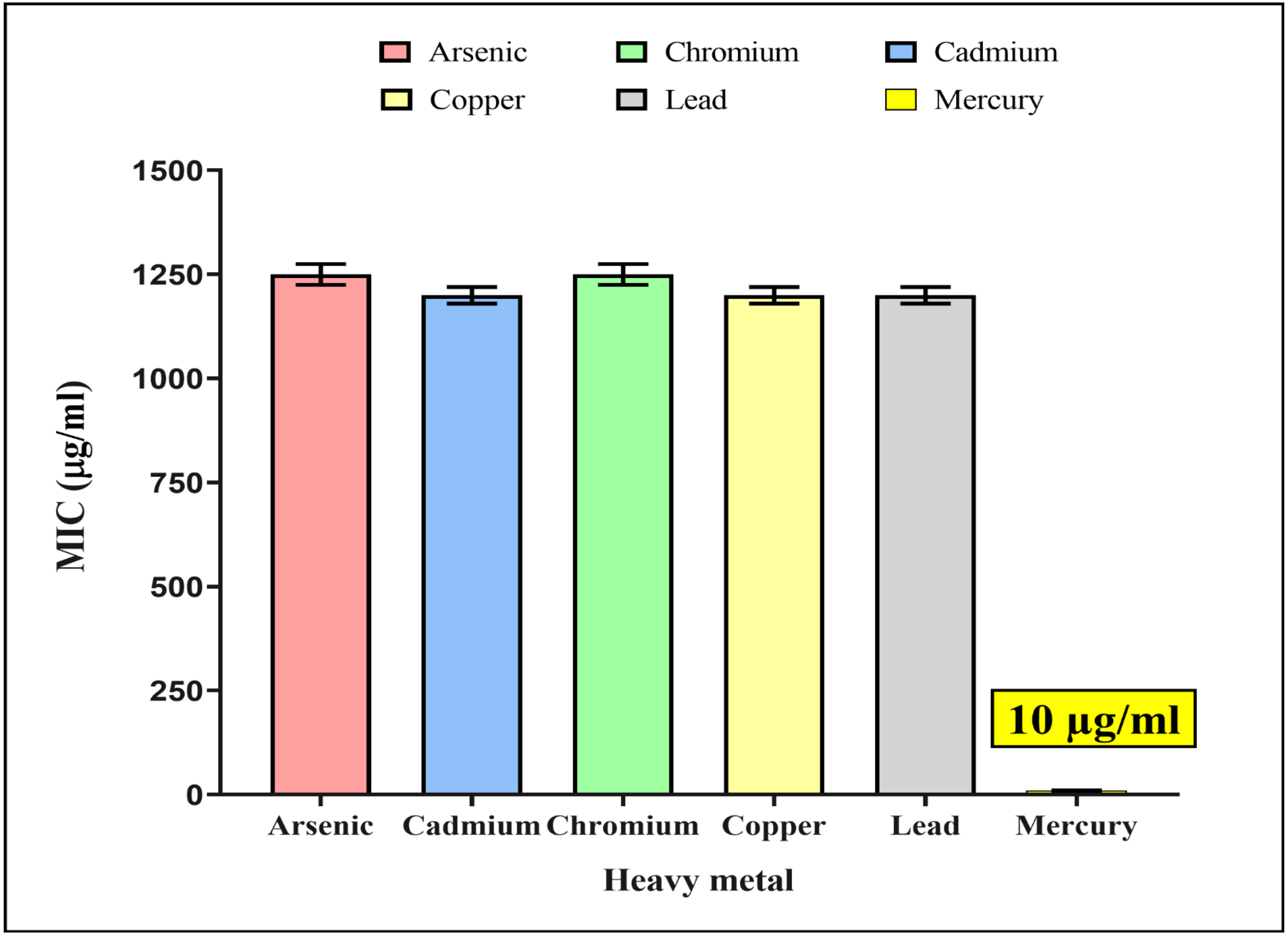

Supplementary figure. S19. Heavy metal resistant/tolerant potentiality (MIC) of the gut isolate *A. veronii* CMF

Supplementary Table

Supplementary Table. S1. Antifungal enzyme production potentiality of *A. veronii* CMF

Gut Symbiont	Antifungal Enzymes		
CMF	Chitinase production (U/ml)	Protease production (U/ml)	β-glucanase production (U/ml)
	22.14±2.12	16.09±0.476	1.89±0.046

Supplementary Table. S2. Composition of antifungal enzyme (chitinase, protease, and β-glucanase) producing agar media

Ingredients	Quantity (g/L)
**Chitinase agar medium**	
Na_2HPO_4	6.00
KH_2PO_4	3.00
NH4Cl	1.00
NaCl	0.5
Yeast Extract	0.05
Chitin	10.00
Final pH	7.0± 0.2
Agar agar	20.00
**Protease agar medium**	
Peptone	5.0
Gelatin	4.0
Beef Extract	3.0
Final pH	7.0± 0.2
Agar agar	20.00
**β-glucanase agar medium**	
K_2HPO_4	1.00
$NaNO_3$	3.00
KCl	0.5
$MgSO_4$, $7H_2O$	0.5
$FeSO_4$, $7H_2O$	0.5
Laminarin	10.00
Final pH	7.0± 0.2
Agar agar	20.00

Supplementary Table. S3. Template sequences of antifungal enzymes in SWISS MODEL

Gut bacteria	Antifungal enzyme		Selected templates	Sequence identity (%)
A. veronii CMF	Chitinase	GH 18 chitinases (MXV30556.1)	2wm0.1.A	55.25
		GH 18 chitinases (MXV29181.1)	4hme.1.B	63.58
		GH 18 chitinases (MXV28648.1)	4txg.1.A	71.93
		GH 19 chitinases (MXV28646.1)	1wvu.1.A	38.30
		GH 19 chitinases (MXV31165.1)	1cns.1.A	28.29
		Chitobiase (MXV29488.1)	3h7l.1.A	55.22
		Chitin disaccharide deacetylase (MXV28820.1)	2i5i.1.A	41.49

Supplementary Table. S4. PMDB accession number of chitinases from *A. veronii* CMF genome

Chitinase	PMDB accession number
GH 18 family chitinases (MXV30556.1)	PM0084140
GH 18 family chitinases (MXV29181.1)	PM0084138
GH 18 family chitinases (MXV28648.1)	PM0084136
GH 19 family chitinases (MXV28646.1)	PM0084135
GH 19 family chitinases (MXV31165.1)	PM0084141
Chitobiase (MXV29488.1)	PM0084139
Chitin disaccharide deacetylase (MXV28820.1)	PM0084137

Supplementary Table. S5. Biocontrol index of the *A. veronii* CMF against fungal pathogens

Fungal Pathogen	Bacterial colony diameter (cm)	Zone diameter (cm)	Biocontrol index
A. alternata	0.94±0.05	6.10±0.20	6.44±0.16
F. oxysporium	1.11±0.06	2.10±0.11	1.9±0.08
F. solani	1.20±0.06	5.86±0.15	4.86±0.14
C. herbarum	1.28±0.05	4.64±0.12	3.63±0.11
R. stolonifer	1.15±0.04	4.25±0.10	3.7±0.11
A. niger	1.19±0.05	6.23±0.21	5.17±0.12
Penicillium sp.	1.1±0.06	2.75±0.11	2.5±0.08
P. digitatum	0.95±0.05	3.33±0.12	3.49±0.09
Paecilomyces sp.	1.26±0.07	5.12±0.14	4.07±0.10
Mucor sp.	1.18±0.06	2.73±0.10	2.31±0.06
C. albicans	0.95±0.06	5.8±0.14	6.1±0.17
C. tropicalis	1.25±0.07	5.13±0.14	4.10±0.12
A. parasiticus	1.2±0.06	3.9±0.11	3.25±0.08
A. flavus	1.15±0.05	5.36±0.16	4.66±0.13
A. fumigatus	1.22±0.07	5.05±0.13	4.14±0.11
T. rubrum	0.95±0.05	2.95±0.12	3.1±0.09
M. gypseum	1.1±0.06	5.02±0.13	4.56±0.11

Supplementary Table. S6. Genomic information from *A. veronii* CMF genome related to Plant growth promoting attributes

Locus tag	Product name	PANNZER annotation	Molecular function	Biological process	Predicted Gene Ontology (GO)	Protein length (aa)
**IAA production**						
Prokka_00164	Anthranilate synthase component 1 TRPE	Anthranilate synthase component 1	Anthranilate synthase activity	Tryptophan biosynthetic process	GO:0004049	544
Prokka_00165	Aminodeoxychorismate/anthranilate synthase component II TRPG	anthranilate synthase	Anthranilate synthase activity	tryptophan biosynthetic process	GO:0004048	199
Prokka_00166	Anthranilate phosphoribosyltransferase TrpD	Anthranilate phosphoribosyltransferase	Anthranilate phosphoribosyltransferase activity	Tryptophan biosynthetic process	GO:0004048	342
Prokka_00167	Bifunctional indole-3-glycerol-phosphate synthase TrpC/phosphoribosylanthranilate isomerase TrpF	Bifunctional indole-3-glycerol-phosphate synthase TrpC/phosphoribosylanthranilate isomerase TrpF	Indole-3-glycerol-phosphate synthase activity	Tryptophan biosynthetic process	GO:0004425	502
Prokka_00168	Tryptophan synthase subunit beta TrpB	Tryptophan synthase beta chain	Tryptophan synthase activity	Tryptophan metabolic process	GO:0004834	397
Prokka_00169	Tryptophan synthase subunit alpha TrpA	Tryptophan synthase alpha chain	Tryptophan synthase activity	Tryptophan metabolic process	GO:0004834	268
Prokka_01319	Tryptophanase	Tryptophanase	Tryptophanase activity	Tryptophan metabolic process	GO:0009034	461
Prokka_01364	Aminodeoxychorismate synthase component I	aminodeoxychorismate synthase	4-amino-4-deoxychorismate synthase activity	Folic acid-containing compound biosynthetic process	GO:0046820	427
Prokka_02440	Aminodeoxychorismate synthase component II	Aminodeoxychorismate/anthranilate synthase component II	4-amino-4-deoxychorismate synthase activity	Tetrahydrofolate biosynthetic process	GO:0004049	193

**Phosphate solubilization**						
Prokka_02935	6-phosphogluconate dehydrogenase, decarboxylating	6-phosphogluconate dehydrogenase	Oxidoreductase activity	Organic acid metabolic process	GO:0016491	292
Prokka_00157	Bifunctional isocitrate dehydrogenase kinase/phosphatase	Isocitrate dehydrogenase kinase/phosphatase	Protein serine kinase activity	Glucose metabolic process	GO:0008772	575
Prokka_00273	Histidine phosphatase family protein	Histidine-type phosphatase	Inositol phosphate phosphatase activity	Phosphoric Monoester Hydrolases	GO:0003993	499
Prokka_00308	Phosphate regulon sensor histidine kinase PhoR	Phosphate regulon sensor protein PhoR	Phosphoprotein phosphatase activity	Cellular response to phosphate starvation	-	431
Prokka_00546	Acylphosphatase	Acylphosphatase	Acylphosphatase activity	Hydrolases	GO:0003998	90
Prokka_03258	Acid phosphatase AphA	Class B acid phosphatase	Acid phosphatase activity	Transferase activity	GO:0016740	239
Prokka_02027	Phosphatase PAP2 family protein	Undecaprenyl-diphosphate phosphatase	Acid phosphatase activity	Transferase activity	GO:0003993	167
Prokka_01550	Hypothetical protein	Alkaline phosphatase	Arylsulfatase activity	Hydrolysis of aromatic esters	GO:0004065	616
Prokka_01554	Phosphoenolpyruvate synthase	Phosphoenolpyruvate synthase	Pyruvate metabolic process	Gluconeogenesis	GO:0008986	790
Prokka_01655	Alkaline phosphatase	Alkaline phosphatase	Alkaline phosphatase activity	Carbohydrate metabolic process	GO:0004035	456
Prokka_01656	Sulfatase-like hydrolase/transferase	Alkaline phosphatase	Alkaline phosphatase activity	Carbohydrate metabolic process	GO:0004035	463
Prokka_01696	Exopolyphosphatase	Exopolyphosphatase	Exopolyphosphatase activity	Polyphosphate catabolic process	GO:0004309	496
Prokka_01782	Phosphoenolpyruvate carboxylase	Phosphoenolpyruvate carboxylase	Phosphoenolpyruvate carboxylase activity	Oxaloacetate metabolic process	GO:0008964	877
Prokka_01990	Alkaline phosphatase family protein	Alkaline phosphatase family protein	Hydrolase activity	Phosphate containing compound metabolic process	GO:0016787	277
Prokka_02447	Beta-phosphoglucomutase family hydrolase	Predicted phosphatase/hydrolase, CbbY family	Sugar-phosphatase activity	Phosphate metabolic Process	GO:0050308	200
Prokka_02665	Phosphatase	Phosphatase	Phosphatase activity	Phosphorus metabolism	GO:0016791	246

Prokka_02764	Protein phosphatase CheZ	Protein phosphatase CheZ	Phosphoprotein phosphatase activity	Phosphoprotein phosphatase activity	-	246
Prokka_02871	Beta-phosphoglucomutase family hydrolase	Beta-phosphoglucomutase family hydrolase	Sugar-phosphatase activity	Mannosyltransferase activity	GO:0050308	196
Prokka_03014	Sugar-phosphatase	Sugar-phosphatase	Phosphatase activity	Phosphate-containing compound metabolic process	GO:0016791	269
Prokka_03258	Acid phosphatase AphA	Class B acid phosphatase	Acid phosphatase activity	Phosphorus metabolic process	GO:0003993	239
**Siderophore production**						
Prokka_00118	Ferric enterobactin transport protein FepE	O-antigen chain length regulator	Protein tyrosine kinase activity	Lipopolysaccharide biosynthetic process	GO:0004713	356
Prokka_00846	Siderophore-interacting protein	Side tail fiber protein (Fragment)	Oxidoreductase activity	-	GO:0016491	267
Prokka_00847	TonB-dependent receptor	TonB-dependent receptor	Siderophore uptake transmembrane transporter activity	Siderophore-dependent iron import into cell	-	655
Prokka_00848	Iron ABC transporter permease	Iron-enterobactin transporter subunit membrane component of ABC superfamily	Transmembrane transporter activity	Transmembrane transport	-	35
Prokka_00849	Iron ABC transporter permease	Iron-enterobactin transporter subunit membrane component of ABC superfamily	Transmembrane transporter activity	Siderophore-dependent iron import into cell	-	338
Prokka_00850	Iron ABC transporter permease	Amonabactin ABC transporter permease subunit 1	Transmembrane transporter activity	Siderophore-dependent iron import into cell	-	345
Prokka_00851	ABC transporter ATP-binding protein	Iron-dicitrate transporter subunit ATP-binding component of ABC superfamily KpLE2 phage-like element	ATP hydrolysis activity	Iron ion transport	-	267
Prokka_00852	Iron-siderophore ABC transporter substrate-binding protein	Iron-siderophore ABC transporter substrate-binding protein	Iron coordination entity transport	Outer membrane-bounded periplasmic space	-	305
Prokka_01110	TonB-dependent hemoglobin/transferrin/lactoferrin family receptor	Ligand-gated channel protein	Siderophore uptake transmembrane transporter activity	Siderophore-dependent iron import into cell	-	712
Prokka_01735	Amino acid adenylation domain-containing protein	Non-ribosomal peptide synthetase	2,3-dihydroxybenzoate-serine ligase activity	Enterobactin biosynthetic process	GO:0047527	1329

Prokka_01736	2,3-dihydro-2,3-dihydroxybenzoate dehydrogenase	2,3-dihydro-2,3-dihydroxybenzoate dehydrogenase	2,3-dihydro-2,3-dihydroxybenzoate dehydrogenase activity	Siderophore biosynthetic process	GO:0008667	255
Prokka_01740	TonB-dependent receptor	TonB-dependent receptor	Siderophore uptake transmembrane transporter activity	Siderophore transmembrane transport	-	650
Prokka_01953	TonB-dependent siderophore receptor	Ligand-gated channel protein	Siderophore uptake transmembrane transporter activity	Siderophore transmembrane transport	-	680
Prokka_02011	Ligand-gated channel protein	Ligand-gated channel protein	Siderophore uptake transmembrane transporter activity	Siderophore transmembrane transport	-	650
Prokka_02297	Iron ABC transporter permease	Hemin ABC transporter permease	Transmembrane transporter activity	Siderophore-dependent iron import into cell	-	343
Prokka_02304	TonB-dependent hemoglobin/transferrin/lactoferrin family receptor	TonB-dependent hemoglobin/transferrin/lactoferrin family receptor	Heme transmembrane transporter activity	Siderophore transmembrane transport	-	701
Prokka_03005	TonB-dependent copper receptor	TonB-dependent copper receptor	Siderophore uptake transmembrane transporter activity	Siderophore transmembrane transport	-	655
Prokka_03814	Fe (3^{+})-hydroxamate ABC transporter permease FhuB	Fe (3^{+})-hydroxamate ABC transporter permease FhuB	Siderophore-dependent iron import into cell	Transmembrane transport	-	660
Prokka_03815	ABC transporter substrate-binding protein	ABC transporter substrate-binding protein	Siderophore uptake transmembrane transporter activity	Iron coordination entity transport	-	301
Prokka_03816	ATP-binding cassette domain-containing protein	ABC-type hydroxamate-type ferric siderophore transporter ATP binding protein	ATP hydrolysis activity	Iron ion transport	-	254
Prokka_03818	TonB-dependent siderophore receptor	Iron (III) compound receptor	Siderophore uptake transmembrane transporter activity	Siderophore transmembrane transport	-	698
Prokka_03845	TonB family protein	Energy transducer TonB	Energy transducer activity	Siderophore transport	-	220
**Nitrogen fixation**						
Prokka_00144	Hypothetical protein	Dinitrogenase iron-molybdenum cofactor biosynthesis domain-containing protein	Nitrogen fixation	Cellular process	-	193

Prokka_00706	Glycosyltransferase family 4 protein	Glycosyltransferase SypJ	Nitrogenase activity	Glycosyltransferase activity	GO:0016163	378
Prokka_03423	AAA family ATPase	AAA family ATPase	Nitrogenase activity	-	GO:0016163	215
Prokka_00451	NADH:ubiquinone reductase (Na(+)-transporting) subunit D	Na (+)-translocating NADH-quinone reductase subunit D	oxidoreductase activity, acting on NAD(P)H, quinone or similar compound as acceptor	Nitrogen fixation	GO:0016655	210
Prokka_03396	Nitrogen regulation protein NR(I)	DNA-binding transcriptional regulator NtrC	Phosphorelay response regulator activity	Nitrogen fixation	-	472
Prokka_03397	Nitrogen regulation protein NR(II)	Sensory histidine kinase/phosphatase NtrB	Phosphorelay sensor kinase activity	Nitrogen fixation	GO:0016787	351
**Biofilm formation**						
Prokka_00084	Diguanylate cyclase	Diguanylate cyclase	Hydrolase activity	Cell adhesion involved in single-species biofilm formation	GO:0016779	820
Prokka_00114	Hypothetical protein	Capsule biosynthesis GfcC family protein	-	-	-	248
Prokka_00115	YjbF family lipoprotein	Lipoprotein YmcC	-	-	-	223
Prokka_00194	Exopolysaccharide biosynthesis protein	Exopolysaccharide biosynthesis protein	-	Intracellular signal transduction	-	141
Prokka_00229	GGDEF domain-containing protein	Diguanylate cyclase	Diguanylate cyclase activity	Cell adhesion involved in single-species biofilm formation	GO:0052621	310
Prokka_00278	Diguanylate cyclase	Diguanylate cyclase activity	Kinase activity	Cell adhesion involved in single-species biofilm formation	GO:0052621	512
Prokka_00290	GGDEF domain-containing protein	Diguanylate cyclase	Diguanylate cyclase activity	Cell adhesion involved in single-species biofilm formation	GO:0052621	373
Prokka_00349	GGDEF domain-containing protein	Diguanylate cyclase	Diguanylate cyclase activity	Cell adhesion involved in single-species biofilm formation	GO:0052621	300
Prokka_00417	YqcC family protein	YqcC-like domain-containing protein	-	Single-species biofilm formation	-	111

Prokka_00427	GGDEF domain-containing protein	Diguanylate cyclase	GTP binding	Cell adhesion involved in single-species biofilm formation	GO:0052621	538
Prokka_00572	Sensor domain-containing diguanylate cyclase	Sensory box/GGDEF domain protein	Diguanylate cyclase activity	Cell adhesion involved in single-species biofilm formation	GO:0052621	429
Prokka_00669	GGDEF domain-containing protein	GGDEF domain-containing protein	Diguanylate cyclase activity	Cell adhesion involved in single-species biofilm formation	GO:0052621	412
Prokka_00705	Diguanylate cyclase	Diguanylate cyclase	Diguanylate cyclase activity	Negative regulation of bacterial-type flagellum-dependent cell motility	GO:0052621	348
Prokka_01174	Diguanylate cyclase	Diguanylate cyclase	Diguanylate cyclase activity	Cell adhesion involved in single-species biofilm formation	GO:0052621	415
Prokka_01411	Diguanylate cyclase	Diguanylate cyclas	Cyclic-guanylate-specific phosphodiesterase activity	Cell adhesion involved in single-species biofilm formation	GO:0071111	549
Prokka_01663	GGDEF domain-containing protein	GGDEF domain-containing protein	Diguanylate cyclase activity	Cell adhesion involved in single-species biofilm formation	GO:0052621	378
Prokka_01985	Diguanylate cyclase	Diguanylate cyclase	Diguanylate cyclase activity	Cell adhesion involved in single-species biofilm formation	GO:0004185	544
Prokka_01995	Diguanylate cyclase	Diguanylate cyclase	Diguanylate cyclase activity	Cell adhesion involved in single-species biofilm formation	GO:0052621	332
Prokka_02010	Sensor domain-containing diguanylate cyclase	Diguanylate cyclase	Diguanylate cyclase activity	Cell adhesion involved in single-species biofilm formation	GO:0052621	462
Prokka_02282	Diguanylate cyclase	Membrane-associated sensor domain-containing protein	Diguanylate cyclase activit	Cell adhesion involved in single-species biofilm formation	GO:0052621	383
Prokka_02386	Diguanylate cyclase	Diguanylate cyclase	Diguanylate cyclase activity	Cell adhesion involved in single-species biofilm formation	GO:0052621	312

Prokka_02390	Diguanylate cyclase	Diguanylate cyclase	Diguanylate cyclase activity	Cell adhesion involved in single-species biofilm formation	GO:0052621	532
Prokka_02627	Diguanylate cyclase	Diguanylate cyclase	Diguanylate cyclase activity	Cell adhesion involved in single-species biofilm formation	GO:0052621	615
Prokka_02869	Diguanylate cyclase	Diguanylate cyclase	Diguanylate cyclase activity	Cell adhesion involved in single-species biofilm formation	GO:0052621	546
Prokka_02900	Diguanylate cyclase	Diguanylate cyclase	Diguanylate cyclase activity	Cell adhesion involved in single-species biofilm formation	GO:0052621	340
Prokka_03285	GGDEF domain-containing protein	Diguanylate cyclase	Diguanylate cyclase activity	Cell adhesion involved in single-species biofilm formation	GO:0052621	241
Prokka_03287	Exopolysaccharide biosynthesis protein	Exopolysaccharide biosynthesis protein	-	Cell adhesion involved in single-species biofilm formation	-	212
Prokka_03289	GGDEF domain-containing protein	Diguanylate cyclase	Diguanylate cyclase activity	Cell adhesion involved in single-species biofilm formation	GO:0052621	307
Prokka_03325	Diguanylate cyclase	Diguanylate cyclase	Diguanylate cyclase activity	Cell adhesion involved in single-species biofilm formation	GO:0052621	557
Prokka_03355	Diguanylate cyclase	Diguanylate cyclase	Diguanylate cyclase activity	Cell adhesion involved in single-species biofilm formation	GO:0052621	384
Prokka_03488	GGDEF domain-containing protein	Diguanylate cyclase	Diguanylate cyclase activity	Cell adhesion involved in single-species biofilm formation	GO:0052621	489
Prokka_03514	YhcH/YjgK/YiaL family protein	DUF386 domain-containing protein	-	Single-species biofilm formation	-	156
Prokka_03765	Sensor domain-containing diguanylate cyclase	Diguanylate cyclase	Diguanylate cyclase activity	Cell adhesion involved in single-species biofilm formation	GO:0052621	287

Prokka_03872	Sensor domain-containing diguanylate cyclase	Diguanylate cyclase	Diguanylate cyclase activity	Cell adhesion involved in single-species biofilm formation	GO:0052621	337
Prokka_04004	Diguanylate cyclase	Diguanylate cyclase DosC	Diguanylate cyclase activity	Cell adhesion involved in single-species biofilm formation	GO:0052621	360
Prokka_04121	PAS domain S-box protein	Biofilm dispersion protein BdlA	Transmembrane signaling receptor activity	Signal transduction	-	433
**Ethylene biosynthetic pathway**						
Prokka_03226	1-aminocyclopropane-1-carboxylate deaminase/D-cysteine desulfhydrase	1-aminocyclopropane-1-carboxylate deaminase/D-cysteine desulfhydrase	D-cysteine desulfhydrase activity	Phosphoenolpyruvate (PEP) Metabolism	GO:0019148	307
Prokka_03707	Serine dehydratase subunit alpha family protein	L-cysteine desulfidase CyuA	L-cysteine desulfhydrase activity	L-cysteine catabolic process to pyruvate	GO:0080146	429
**Adhesion and attachment**						
Prokka_01610	Flagellin	Flagellin flaA	Structural molecule activity	Bacterial-type flagellum	-	304
Prokka_01852	Flagellin B	Flagellin flaB	Structural molecule activity	Bacterial-type flagellum	-	371
Prokka_00283	Porin OmpA	Major outer membrane protein OmpAI	Porin activity	Transmembrane transport	-	338
Prokka_00285	OmpA family protein	Major outer membrane protein OmpAII	Porin activity	Transmembrane transport	-	345
Prokka_02760	OmpA family protein	Cell envelope biogenesis protein OmpA	-	Transmembrane transport	-	306
Prokka_02761	Flagellar motor protein	Flagellar motor protein MotC	Bacterial-type flagellum-dependent swarming motility	Protein transport	-	245
Prokka_02901	Porin OmpA	Porin OmpA	Porin activity	Transmembrane transport	-	344

Supplementary Table. S7. Genomic information from *A. veronii* CMF genome related to heavy metal remediation

Locus tag	Product name	PANNZER annotation	Molecular function	Biological process	Predicted Gene Ontology (GO)	Protein length (aa)
**Arsenic**						
Prokka_00064	ArsC family reductase	ArsC family reductase	Arsenate reductase (glutaredoxin) activity	-	GO:0008794	116
Prokka_01491	Heavy metal-binding domain-containing protein	Heavy metal-binding domain-containing protein	-	-	-	105
Prokka_01536	Arsenate reductase (glutaredoxin)	Arsenate reductase	Arsenate reductase (glutaredoxin) activity	-	GO:0008794	116
Prokka_00180	Arsenate reductase (glutaredoxin)	Arsenate reductase	Arsenate reductase (glutaredoxin) activity	Response to arsenic-containing substance	GO:0008794	141
Prokka_00181	ACR3 family arsenite efflux transporter	ACR3 family arsenite efflux transporter	Arsenite transmembrane transporter activity	Arsenite transport	-	354
Prokka_00182	Arsenical pump-driving ATPase	Arsenical pump-driving ATPase	ATPase-coupled arsenite transmembrane transporter activity	Detoxification of arsenic-containing substance	GO:0015446	587
Prokka_00183	Arsenite efflux transporter metallochaperone ArsD	Arsenite efflux transporter metallochaperone ArsD	DNA binding	Response to arsenic-containing substance	-	122
Prokka_00184	Helix-turn-helix transcriptional regulator	Arsenic operon regulator	DNA-binding transcription factor activity	Regulation of DNA-templated transcription	-	122
Prokka_02007	Periplasmic heavy metal sensor	Response regulator	Phosphorelay response regulator activity	Phosphorelay signal transduction system	-	116
**Cadmium**						
Prokka_01431	Cadmium-translocating P-type ATPase	Cadmium-translocating P-type ATPase	P-type divalent copper transporter activity	Copper ion transmembrane transport	GO:0043682	794
Prokka_01508	Cation diffusion facilitator family transporter	Cation diffusion facilitator family transporter	Cadmium ion transmembrane transporter activity	Cadmium ion transmembrane transport	-	308
Prokka_02511	Zinc/cadmium/mercury/lead-transporting ATPase	Zinc/cadmium/mercury/lead-transporting ATPase	Cadmium ion transmembrane transporter activity	Cadmium ion transmembrane transport	-	811

Prokka_02607	Divalent metal cation transporter	Divalent metal cation transporter MntH	Cadmium ion transmembrane transporter activity	Cadmium ion transmembrane transport	-	407
Prokka_03312	Cation transporter	Cation transporter	Cadmium ion transmembrane transporter activity	Cadmium ion transmembrane transport	-	300
Prokka_03353	Cadmium-translocating P-type ATPase	Heavy metal translocating P-type ATPase	P-type divalent copper transporter activity	Copper ion transmembrane transport	-	810
Prokka_03992	Cation diffusion facilitator family transporter	Divalent metal cation transporter FieF	Cadmium ion transmembrane transporter activity	Cadmium ion transmembrane transport	-	303
**Chromium**						
Prokka_03604	Chromate efflux transporter	Chorismate-binding protein	Chromate transmembrane transporter activity	Chromate transport	-	388
**Copper**						
Prokka_00303	Copper homeostasis protein CutC	Copper homeostasis protein CutC	Copper ion binding	-	-	241
Prokka_00853	Copper-binding protein	Secretion protein HlyD	-	-	-	117
Prokka_00854	Efflux RND transporter permease subunit	HAE1 family transport protein (Probable substrate copper)	Xenobiotic transmembrane transporter activity	Xenobiotic transport	-	1042
Prokka_00855	Efflux RND transporter periplasmic adaptor subunit	Efflux RND transporter periplasmic adaptor subunit	Transition metal ion binding	Transmembrane transporter activity	-	510
Prokka_00866	Azurin	Azurin	Copper ion binding	-	-	146
Prokka_02880	Copper chaperone PCu(A)C	Copper chaperone PCu(A)C	-	-	-	149
Prokka_02932	Thioredoxin domain-containing protein	Copper-sensitivity protein C	Disulfide oxidoreductase activity	-	-	246
Prokka_03354	Cu(I)-responsive transcriptional regulator	Transcriptional regulator of copper-responsive regulon genes	Copper ion binding	Positive regulation of DNA-templated transcription	-	129
Prokka_03931	Divalent-cation tolerance protein CutA	Divalent-cation tolerance protein CutA	Copper ion binding	Response to metal ion	-	105
**Lead**						
Prokka_02511	Zinc/cadmium/mercury/lead-transporting ATPase	Zinc/cadmium/mercury/lead-transporting ATPase	Cadmium ion transmembrane transporter activity	Cadmium ion transmembrane transport	-	811
**Mercury**						
Prokka_00007	Pyridine nucleotide-disulfide oxidoreductase	Pyridine nucleotide-disulfide oxidoreductase	Mercury (II) reductase ($NADP^+$) activity	-	GO:0016152	717

Supplementary Table. 8. Fungal strains used in this study

	Fungal strains	Source	Growth conditions	Properties
**Plant Pathogenic Fungi**	*Alternaria alternata* VBAV007	Lab isolate	ME, 28ºC	Leaf spot disease of *Aloe vera*
	Fusarium oxysporum MTCC2480	MTCC	ME, 28ºC	Pathogen of wilt disease of pea
	Fusarium solani	Lab isolate	ME, 28ºC	Black root rot disease
	Colletotrichum acutatum MTCC2074	MTCC	ME, 28ºC	Pathogen of blight of banana
	Cladosporium herbarum (MTCC 2143)	MTCC	ME, 28ºC	Leaf spots, scab, and postharvest rots
	Rhizopus stolonifer VBAM1	Lab isolate	ME, 28ºC	Pathogen of fruit rot of jack fruit
	Aspergillus niger VBS1	Lab isolate	ME, 28ºC	Black rot of onion and garlic
	Penicillium digitatum VBCS1	Lab isolate	ME, 28ºC	Orange spoilage
	Paecilomyces sp. VBSD 13	Lab isolate	ME, 28ºC	Post-harvest disease in apple
**Animal Pathogenic Fungi**	*Candida albicans* MTCC183	MTCC	ME, 28ºC	Human pathogen
	Candida tropicalis MTCC184	MTCC	ME, 28ºC	Human pathogen
	Aspergillus parasiticus MTCC2796	MTCC	ME, 28ºC	Toxin (Aflatoxin) producer
	Aspergillus flavus MTCC2799	MTCC	ME, 28ºC	Toxin (Aflatoxin) producer
	Aspergillus fumigatus MTCC2550	MTCC	ME, 28ºC	Antigen producer
	Trichophyton rubrum MTCC296	MTCC	ME, 28ºC	Skin disease (pigment formation)
	Microsporum gypseum MTCC2819	MTCC	ME, 28ºC	Skin disease (degradation of keratin)
**Spoilage Fungi**	*Mucor* sp. VBBM7	Lab isolate	ME, 28ºC	Bread spoilage

- ME = Malt Extract Agar
